# Prognostic value and immune-infiltration pattern of FOXD3-AS1 in patients with glioma

**DOI:** 10.3389/fphar.2023.1162309

**Published:** 2023-04-04

**Authors:** Zhenhua Chen, Yi Zhang, Sujuan Feng, Jiaqi Yuan, Dongliang Shi, Yong Wang, Yongdong Li, Jun Dong

**Affiliations:** ^1^ Department of Neurosurgery, Second Affiliated Hospital of Soochow University, Suzhou, China; ^2^ Department of Neurosurgery, Affiliated Hospital 2 of Nantong University and Affiliated Hospital of Kangda College of Nanjing Medical University, Nantong, China; ^3^ Department of Nephrology, Affiliated Hospital 2 of Nantong University and Affiliated Hospital of Kangda College of Nanjing Medical University, Nantong, China; ^4^ Department of Neurosurgery, Joint Logistics Support Unit No 904 Hospital, Wuxi, China

**Keywords:** FOXD3-AS1, prognosis, immune infiltration, oxidative stress, TCGA

## Abstract

Gliomas are difficult-to-treat brain tumors due to their aggressive nature, rapid proliferation, and high invasiveness (Zhang et al., J Cell Biochem, 2019, 120 (9), 15106–15118; Ge et al., Int J Biochem Cell Biol, 2021, 139, 106054). FOXD3-AS1 has been identified as an emerging potential target for tumor prediction and treatment in many studies (Qin et al., Front Oncol, 2021, 11, 688027). However, the utility of FOXD3-AS1 has not been reported in glioma patients (Li et al., Cancer Manag Res, 2021, 13, 9037–9048). The differential profiles of FOXD3-AS1 in TCGA–GBMLGG database were analyzed across clinical subgroups. The analysis of overall survival (OS), disease-specific survival (DSS), and progression-free interval (PFI) revealed that a high level of FOXD3-AS1 was associated with a poor prognosis and survival outcome. Based on the Cox regression analysis, FOXD3-AS1 was found to be a high-risk factor for glioma that affects prognosis outcomes independently. More importantly, because oxidative stress is closely linked to glioma prognosis, we focused on the potential mechanisms of six oxidative stress co-expressed genes with FOXD3-AS1. In addition, the predictive value of FOXD3-AS1 was determined for each clinical subgroup status. The ROC curve results showed that FOXD3-AS1 had a good predictive performance. A stratified clinicopathological subgroup analysis revealed that high expression of FOXD3-AS1 is associated with a poor prognosis. This also indicates a link between FOXD3-AS1 and tumorigenesis and prognosis, which has potential application value. Furthermore, the immune cell infiltration of FOXD3-AS1 and the signal marker correlation suggested that immune cell infiltration differed significantly between immune cell subsets. To the best of our knowledge, this is the first report to investigate FOXD3-AS1 in glioma and how it may modulate GBM and LGG immune microenvironments. Furthermore, FOXD3-AS1 was detected in tumor and paraneoplastic tissues using RT–qPCR. Transwell analysis verified the migration and invasion of the FOXD3-AS1 knockout group *in vitro* to a certain extent. In conclusion, FOXD3-AS1 can be used as a prognostic indicator for GBM and LGG, and it is closely related to immune infiltration and response to oxidative stress, which may contribute to the advancement of glioma immunotherapy research.

## Introduction

Gliomas account for more than 70% of malignant brain tumors, with glioblastoma multiforme (GBM) and low-grade gliomas (LGGs) being the most prevalent (Zhang et al., 2019). Based on the degree of malignancy of the disease, the WHO classified gliomas as grades I–IV5, David et al. (2021). Simultaneously, the WHO included molecular characteristics of primary brain tumors, such as chromosome 19q and 1p deletions and isocitrodehydrogenase (IDH), as reference variables for glioma prognosis based on the molecular mechanisms of glioma pathogenesis and progression (Quinn et al., 2014). Although the curative effect of traditional anti-tumor therapies recommended by the guideline has improved in glioma patients, the results are still not encouraging. Immunotherapy has gradually become an important treatment option for many solid tumors (Cugurra et al., 2021). However, immune checkpoint inhibitors are ineffective in GBM and LGG (Zhang et al., 2022). Tumor heterogeneity is a major risk factor for recurrence (James and Howard, 2021). As one of the hub factors, the microenvironment where immune cells infiltrate has a significant impact on the therapy of malignant glioma (Sean and Marc, 2013). Therefore, it is critical to focus on specific molecular and microenvironmental properties and to identify biological markers that affect GBM and LGG metabolism to provide a foundation for more targeted potential therapies (Aaron et al., 2015).

The lncRNA forkhead box D3 antisense RNA 1 (FOXD3-AS1) has been linked to cancer progression (Liu et al., 2022; Yao et al., 2022; Zhang et al., 2022). FOXD3-AS1 is a key regulator of cancer that is significantly upregulated in melanoma cells, promoting their proliferation and migration. FOXD3-AS1 knockdown can promote melanoma cell apoptosis. It was also found that miR-127-3p is negatively regulated after binding to FOXD3-AS1, implying that FOXD3-AS1 is involved in melanoma progression *via* the miR-127-3p/FJX1 axis (Ge et al., 2021). FOXD3-AS1 expression was significantly increased in cervical cancer tissues and cell lines. In contrast, competitive spongization of miR-128-3p increased LIMK1 expression, resulting in poor tumor differentiation, increased tumor size, and positive lymph node metastasis (Qin et al., 2021). FOXD3-AS1 expression is increased in ischemic cardiomyocyte injury. FOXD3-AS1 binds directly to miR-765 as a cRNA, regulating BCL2L13 expression. This pathway can serve as a key signaling pathway that regulates cerebral ischemia-reperfusion injury (Chenwei et al., 2021). Several studies have shown that the immune microenvironment in tumors is regular and that oxidative stress plays an important role in the tumor microenvironment (Wencke et al., 2015).

Glioma has been linked to various molecular events associated with the oxidative stress pathway (Bo et al., 2016). Specifically, during glioma progression, glioma stem cells (GSCs) may be influenced by their enrichment in hypoxic microenvironments with significantly elevated levels of oxidative stress, leading to tumor initiation and proliferation, therapeutic resistance, and subsequent tumor recurrence. However, there are some paradoxical claims that GSCs produce less reactive oxygen species (ROS) in the tumor mass, thereby increasing the levels of oxidative stress chemically (Sung-Hak et al., 2014; Meyfroidt et al., 2017).

Several studies have investigated the role of FOXD3-AS1 in cancer. Here, we investigated the role of the immune system in the expression of FOXD3-AS1 in glioma (Wu et al., 2019; Sun et al., 2020; Li et al., 2021b; Li et al., 2021a). The relationship between FOXD3-AS1 and immune cells was analyzed in TCGA (The Cancer Genome Atlas) database using CIBERSORT and TIMER. The predictive value of FOXD3-AS1 for the 1, 3, and 5-year survival status of GCB and LGG patients was analyzed based on the overall survival (OS). Cox regression analysis revealed that FOXD3-AS1 is an independent risk factor for OS in glioma. A nomogram was established based on FOXD3-AS1 expression. In addition, the differences in immune infiltration between glioma patients with varying levels of FOXD3-AS1 expression were investigated. CIBERSORT analysis revealed that FOXD3-AS1 was positively correlated with a variety of immune cells (Jin et al., 2020). High expression of FOXD3-AS1 was associated with poor OS, disease-specific survival (DSS), and progression-free interval (PFI) in GBM and LGG. We conclude that FOXD3-AS1 can be used for promising the most closely relationship to immune infiltration and oxidative stress in LGG and GBM.

## Materials and methods

### Data acquisition and collation

We downloaded a uniformly pan-cancer dataset from UCSC (http://genome.ucsc.edu) database (Ann. et al., 2008; W James et al., 2002), TCGA Pan-Cancer (https://www.cancer.gov/about-nci/organization/ccg/research/structural-genomics/tcga), from which we further extracted the expression data of ENSG00000230798(FOXD3-AS1) gene in each sample, and further we screened the sample source. We from the previous studies for each tumor mRNA characteristics.

### Searching and screening of phenotypic gene sets

We searched for HALLMARK gene sets in humans with the term “Oxidative stress” in the “GSEA-msigdb” database and obtained a set of “Hallmark” Genes containing 195 genes encoding from the Molecular Signatures Database (MSigDB) (https://www.gsea-msigdb.org/gsea/msigdb) (Ge et al., 2021). The gene set contains genes that encode proteins involved in oxidative phosphorylation.

### GEPIA investigation on FOXD3-AS1 expression difference and survival analysis

We evaluated the relationship between FOXD3-AS1 expression and survival and prognosis of glioma patients from Gene Expression Profile Interaction Analysis (GEPIA) (http://gepia.cancer-pku.cn/) (Chenwei et al., 2021). In addition, the difference of FOXD3-AS1 expression between normal and tumor samples was described by box graph with disease status (normal or tumor) as variable.

### Multivariate and univariate Cox models

Cox analysis were used to evaluate the correlation between FOXD3-AS1 expression and age, sex, IDH mutation, pathological type of glioma, 1p/19q co deletion, treatment response and total survival (OS).

### Enrichment analysis

Correlation genes of FOXD3-AS1 were screened using the “limma” package (Matthew et al., 2015). The threshold value is set as |log 2FC|>2, adj. *p* < 0.05. In addition, gene ontology (GO) (http://geneontology.org/) (Wencke et al., 2015) and Kyoto Encyclopedia of Genes and Genomes (KEGG) (https://www.kegg.jp/) (Zhang et al., 2019) analysis use relevant software packages with an accepted threshold.

### Gene set enrichment analysis

We used the computational method gene set enrichment analysis (GSEA) to determine the statistical significance of a preferentially defined set of genes and whether there were consistent differences between the two biological states. In this study, we permuted each gene combination analyzed a thousand times. The FOXD3-AS1 expression level was used as a phenotypic marker. Potential enrichment pathways involved in the gene set “c2. cp.v7.2. symbols.gmt” were retrieved from a molecular marker database; were analyzed with GSEA 4.0.3 (Jin et al., 2020; Sun et al., 2020). In addition, normalized enrichment scores (NESs), nom *p*-values, and FDR q-values of GSEA were generated to separate enrichment pathways into two phenotypes.

### Prognostic analysis

Clinical data were extracted from TCGA database. The clinical features including age, sex p/19q co deletion, IDH mutation, pathological type and grade of glioma were significantly correlated with FOXD3-AS1 as shown in Supplementary Table S1. We determined the median expression of FOXD3-AS1 to create differential groups. Survival analysis showed difference of OS, DSS, and PFI. In addition, univariate and multivariate Cox regression analysis were used to determine independent prognostic variables. We use the package “rms” to generate Nomogram to visualize the prognosis of FOXD3-AS1. ROC and correction curve are used for discrimination and correction.

### Molecular network construction

The expression of genes is interrelated, especially the genes regulating the same biological process. Therefore, in order to reveal the possible molecular interaction network of six oxidative stress co-expression genes closely related to FOXD3-AS1, we predicted through miRNet (https://www.mirnet.ca/) and ENCODE (https://www.encodeproject.org) databases (Martin, 2004). We predict miRNA and transcription factors of hub nodes respectively. After the forecast results are exported, they are processed and plotted by using cycloscape.

### Immune-infiltration pattern

The “Estimation” software package was used to evaluate the tumor purity of 33 human tumors. Specifically, the immune and interstitial scores represent the abundance of immune and interstitial components, respectively. The ESTIMATE score is the sum of previous scores and indirectly represents the purity of the tumor. The correlation of immune scores in pan-cancer were depicted. Tumor Immune Assessment Resource 2.0 Web servers are a comprehensive resource for systematic analysis of immune infiltrates in different cancer types. We investigated differential expression of FOXD3-AS1 in tumors and adjacent normal tissues across all TCGA cohorts by the Timer database.

### Immune deconvolution algorithm

Then, based on several immune deconvolution algorithms, we investigated the relationship between FOXD3-AS1 expression and immune infiltration. To maximize the accuracy of the results, using another method, the r-pack CIBERSORT assessed the level of infiltration of immune cells, based on the LM22 background gene set provided by CIBERSORT (Binbin et al., 2018), the content of 22 immune cells in each sample was calculated to reflect the infiltration level (Aaron et al., 2015). Similarly, for FOXD3-AS1-immune cell pairs with significant correlations, scatter plots were drawn and correlation curves were fitted. Finally, we assessed the association of FOXD3-AS1 with immune cell subsets.

### TIMER database analysis

For the inclusive analysis of the TIICs, TIMER database (http://timer.comp-genomics.org/) was utilized by using the RNA-seq expression profile data (Bo et al., 2016).

### Quantitative reverse transcription-polymerase chain reaction (qRT-PCR)

Total RNA was extracted from normal tissue and tumor tissue from glioma patients using TRIzol reagent (Sigma-Aldrich, United States). Quantitative reverse transcription-polymerase chain reaction (qRT-PCR) was conducted on the obtained RNA from each sample (2 μg) with FastStart universal SYBR ^®^Green Master (Roche, United States) on a LightCycler 480 PCR System (Roche, United States). The cDNA was utilized as a template with a reaction volume of 20 μL (2 μL of cDNA template, 10 μL of PCR mixture, 0.5 μL of forward and reverse primers, and an appropriate water volume). The following procedures were utilized for the PCR reactions: Cycling conditions started with an initial DNA denaturation phase at 95°C for 30 s, followed by 45 cycles at 94°C for 15 s, 56°C for 30 s, and 72°C for 20 s. Three separate analyses were performed on each sample. Based on the 2^−ΔΔCT^ method, data from the threshold cycle (CT) were obtained and standardized to the levels of glyceraldehyde 3-phosphate dehydrogenase (GAPDH) in each sample. The expression levels of mRNA were compared to controls obtained from normal tissues. The following is a list of the sequences of primer pairs for the genes that were being targeted.

**Table:** 

Gene	Forward primer sequence (5–3)	Reverse primer sequence (5–3)
CA3	CACACCGTGGATGGAGTCAA	AGCCCTGGTAGGTCCAGTAG
GATA4	CCTGTCACTCAGACATCGCA	TACGCGGTGATTATGTCCCC
H19	CAAAGCCTCCACGACTCTGT	TGGGGCGTAATGGAATGCTT
HP	GGGACAGCTTTTTGCAGTGG	ACTCCATCTCCTTCTGTGCG
MMP9	TCTATGGTCCTCGCCCTGAA	TTGTATCCGGCAAACTGGCT
FOXD3-AS1	AACAAAGGGACGAGAGACGC	TCTTTAAAGCCGCTCCCTGG
18s	GGCACGTGTGGACCATCTAA	AGGATACACAGCAGCACTGAC
GAPDH	AATGGGCAGCCGTTAGGAAA	GCCCAATACGACCAAATCAGAG

### Cell culture and transient transfection

ATCC (Beijing Beina Chuanglian Biotechnology Institute) provided U-87 and U-251 human glioma cell lines, which were then incubated in F12 and DMEM supplemented with 10% fetal bovine serum (FBS, Gibco, Carlsbad, CA, United States), respectively. Both cell lines were kept in a humidified incubator and maintained at 37°C with 5% carbon dioxide. The negative control (NC) and FOXD3-AS1 siRNA (Thermofisher, United States) were transfected into the glioma cells utilizing Lipofectamine 2000 (Invitrogen, Carlsbad, CA, United States) according to the manufacturer’s guidelines. The target sequences for FOXD3-AS1 siRNAs were 5′-GTG​TGG​ACA​AAT​CCT​CCA​AGA-3′ (FOXD3-AS1 si 1) and 5′-GAG​GAG​TTC​CGA​GAG​GAA ATA-3′ (FOXD3-AS1 si 2). The target sequences for FOXD3-AS1-overexpression were F: 5′-ATA​CTC​GAG​CGA​ACA​AAG​GGA​CGA​GAG​ACG​C-3′, R: 5′-ATA​GCG​GCC​GCT​CTT​TAA​AGC​CGC​TCC​CTG​G-3′ (FOXD3-AS1 oe 1) and F: 5′-ATA​CTC​GAG​CGA​AGA​GTA​AGA​GCA​GCG​CAC​C-3′, R: 5′-ATA​GCG​GCC​GCC​GGG​AAA​GAG​CAG​GTA​GGA​C-3′ (FOXD3-AS1 oe 2). At the same time, cell culture dishes/plates, and centrifuge tubes were obtained from NEST Biotechnology Co. Ltd (Wuxi, China)

### Drugs treatment and CCK8

MMP-9-IN-1 (#HY-135232, MCE, United States) and GATA4-NKX2-5-IN-1 (#HY-103484, MCE, United States) was dissolved in 10% DMSO, 40% PEG300, 5% Tween-80, and 45% saline. As controls, solutions with just 10% DMSO, 40% PEG300, 5% Tween-80, and 45% saline were utilized. MMP-9-IN-1 and GATA4-NKX2-5-IN-1 were administered at a dose of 5 mg/mL once and observed for 72 h. Cells were seeded into a 96-well plate at a density of 1000 cells per well. CCK8 reagent (Beyotime, China) was added into the wells and cells cultured for 1.5 h. The absorbance was determined at 450 nm.

### Transwell assay

Transwell assays for glioma cell (U-87, U-251) migration and invasion were performed. Briefly, cells (5 × 10^4^) were inoculated into chambers coated (for invasion) or uncoated with Matrigel (for migration; BD Biosciences, San Jose, CA). The top layer was added with SFM, whereas the bottom layer was added with a medium entirely composed of DMEM. Following an incubation period of 24 h, migrating or invading cells were dyed with 0.1 percent crystalline violet and subsequently fixed with 4% paraformaldehyde. The counting of the cells was done under a light microscope.

### Statistical analyses

Data are presented as means ± standard error (SD). Differences between groups were analyzed using a Student’s t-test. Statistical analyses were performed using R 4.0.2. *p* < 0.05 (two-tailed) was considered statistically significant.

## Results

### Differential expression of FOXD3-AS1 and its prognostic significance in pan-cancer

**FIGURE 1:**
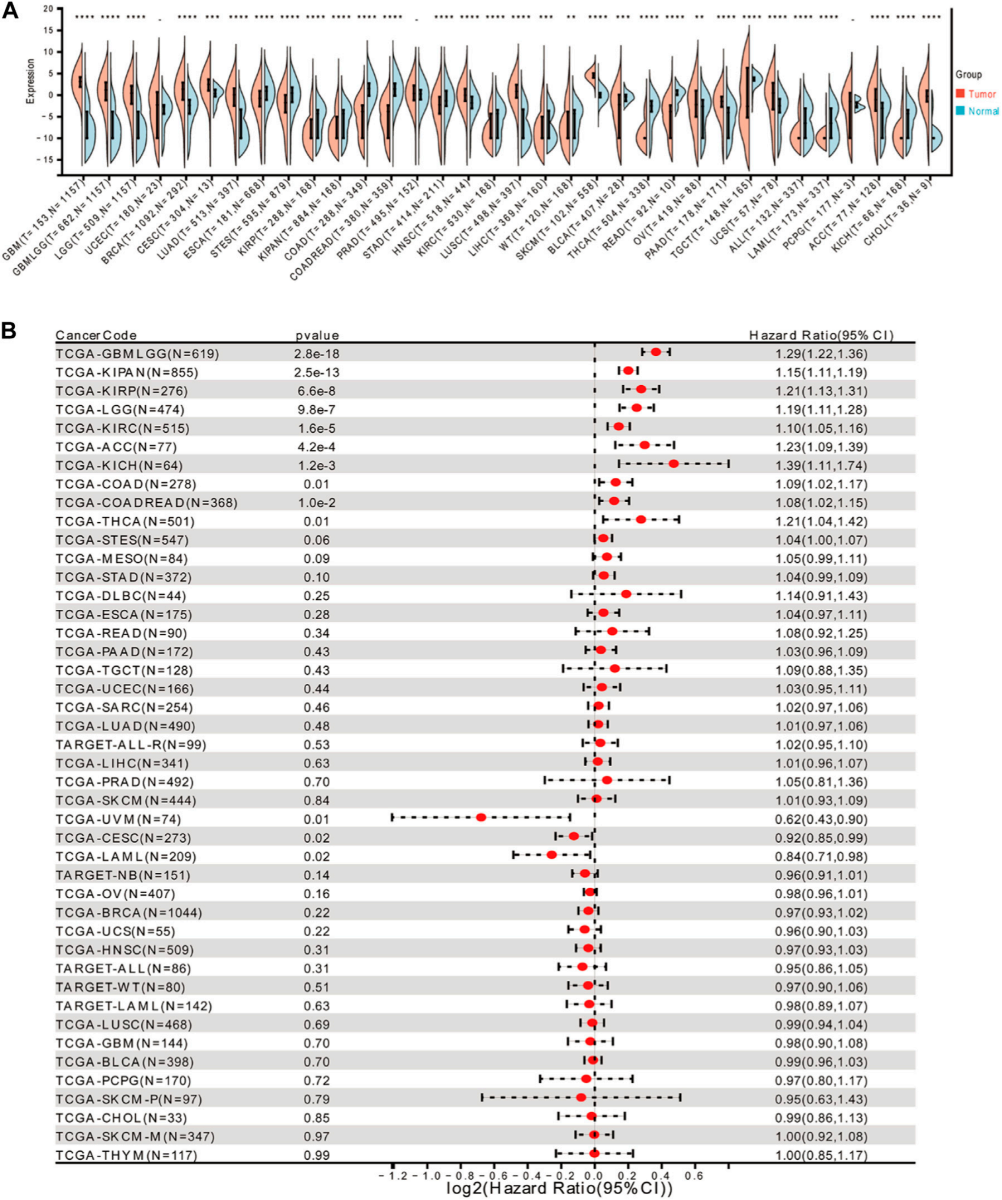
Expression of FOXD3-AS1 in pan-cancer and its prognostic significance. **(A)** Differential expression of FOXD3-AS1 in pan-cancer patients; **(B)** Cox analysis of prognostic forest plot of FOXD3-AS1 expression *versus* survival of TCGA pan-cancer samples. * * *p < 0.01, * * *p < 0.001.

The Wilcoxon rank sum and signed ranks tests were used to compare the expression levels of FOXD3-AS in normal and tumor samples in each tumor. We found significant upregulation in 14 tumors, including GBM (tumor: 2.85 ± 1.90, normal: −7.57 ± 3.46; *p* = 1.6e-108), GBMLGG (tumor: 0.31 ± 3.74, normal: −7.57 ± 3.46; *p* = 6.0e-215), LGG (tumor: −0.45 ± 3.83, normal: −7.57 ± 3.46; *p* = 7.9e-168), and BRCA (tumor: 0.37 ± 3.70, normal: −3.37 ± 3.49, *p* = 1.0e-60). Similarly, significant downregulation was detected in 17 tumors, including ESCA (tumor: −1.52 ± 3.73, normal: 0.28 ± 2.57; *p* = 4.5e-11), STES (tumor: −2.62 ± 3.91, normal: −0.12 ± 2.78; *p* = 1.9e-42), and KIRP (tumor: −8.19 ± 3.18, normal: −5.70 ± 3.21; *p* = 2.8e-14). (Figure 1A). The Cox proportional hazards regression model of pan-cancer based on the coxph function was analyzed. The forest plot was used to calculate the hazard ratio and significance of FOXD3-AS1 in pan-cancer. In general, the relationship between each tumor was investigated, and the logrank test was used to determine the statistical significance of the prognosis. Finally, we observed that in 10 tumor types [TCGA–GBMLGG (N = 619, *p* = 2.8e-18, HR = 1.29 (1.22, 1.36)], TCGA–LGG [*n* = 474, *p* = 9.8e-7, HR = 1.19 (1.11, 1.28)], TCGA–KIRP [*n* = 276, *p* = 6.6e-8, HR = 1.21 (1.13, 1.31)], TCGA–KIPAN [*n* = 855, *p* = 2.5e-13, HR = 1.15 (1.11, 1.19), TCGA–COAD (*n* = 278, *p* = 0.01, HR = 1.09 (1.02, 1.17)] TCGA–COADREAD [*n* = 368, *p* = 1.0e-2, HR = 1.08 (1.02, 1.15)], TCGA–KIRC [*n* = 515, *p* = 1.6e-5, HR = 1.10 (1.05, 1.16)], TCGA–THCA [*n* = 501, *p* = 0.01, HR = 1.21 (1.04, 1.42)], TCGA–ACC [*n* = 77, *p* = 4.2e-4, HR = 1.23 (1.09, 1.39)], TCGA–KICH [*n* = 64, *p* = 1.2e-3, HR = 1.39 (1.11, 1.74)] In 3 tumor types (TCGA–CESC (N = 273, *p* = 0.02, HR = 0.92 (0.85, 0.99)), TCGA–UVM [*n* = 74, *p* = 0.01, HR = 0.62 (0.43, 0.90)] The prognosis of TCGA–LAML [*n* = 209, *p* = 0.02, HR = 0.84 (0.71, 0.98)] was poor (Figure 1B).

### Comprehensive analysis of the FOXD3-AS1 stem cell index in pan-cancer tumor samples

**FIGURE 2:**
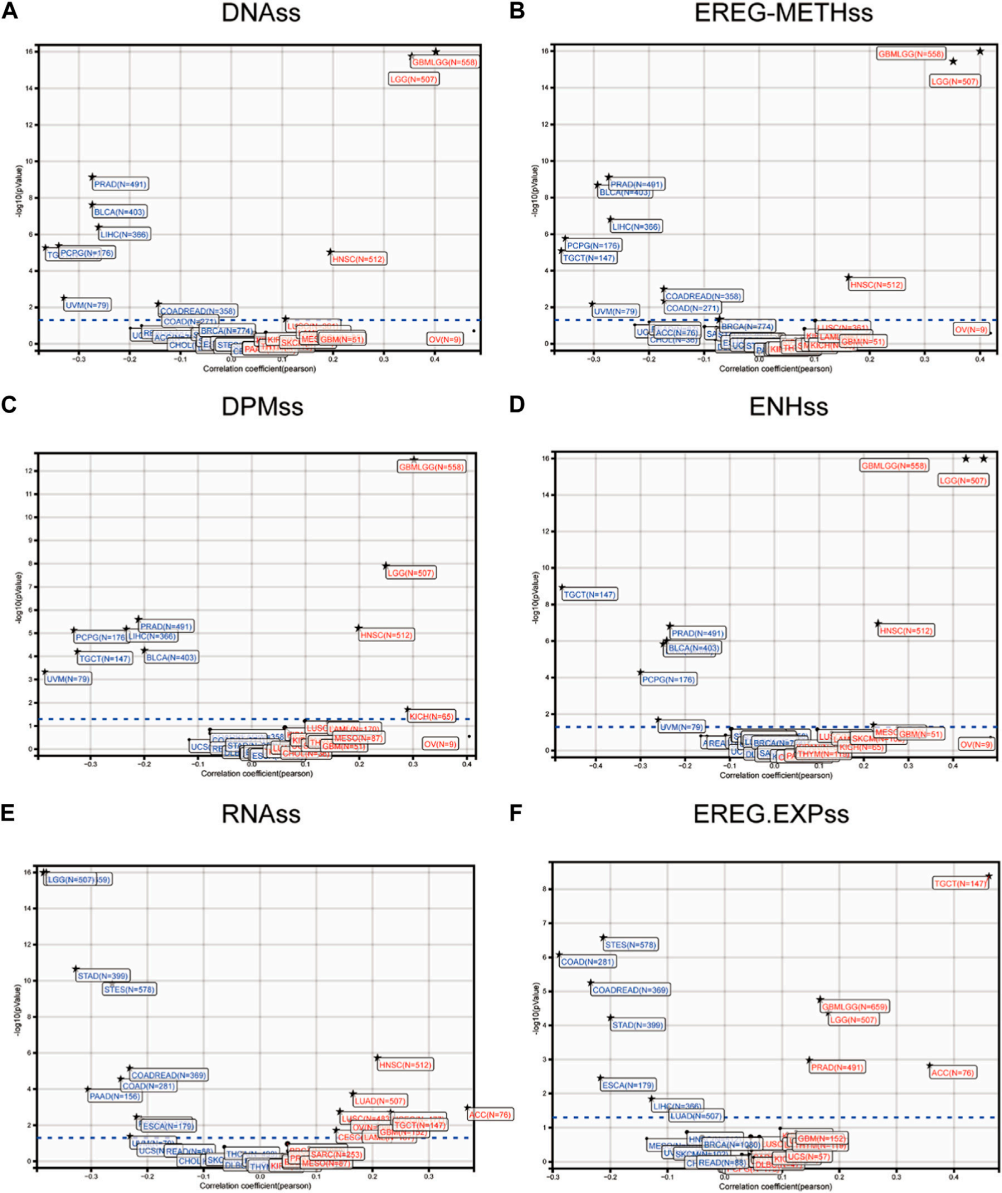
Correlation of stem cell index of FOXD3-AS1 in pan cancer tumor samples. **(A)** Scatter plots show that FOXD3-AS1 and the DNA methylation based Stemness Scores (DNAss), empirically regulated DNA methylation based (EREG-METHss) **(B)**, differentially methylated probes based (DMPss) **(C)**, Enhancer Elements/DNAmplification based (ENHss) **(D)**, RNA expression based score (RNAs) **(E, F)** and Epigenetically regulated RNA expression based (EREG. EXPss).

We obtained six tumor stem cell indices calculated based on mRNA expression and methylation characteristics from previous studies and analyzed their differences between samples to reflect the correlation (Figure 2). Scatter plots depict the correlation between tumor patient samples and FOXD3-AS1 stem cell indices, including different stemness scores (DNA metabolism-based stemness scores (DNAss) and RNA expression-based stemness scores (RNAss)) derived from the stemness group (Figure 2E). DNA methylation-based stem cell signature probes were derived from the main figures in the PanCanAtlas paper. Furthermore, scatter plots of epigenetically regulated DNA methylation-based stemness scores (EREG-METHss) (Figure 2B), differently methylated probe-based stemness scores (DMPss) (Figure 2C), enhancer elements/DNA amplification-based (ENHss) (Figure 2D), and epigenetically regulated RNA expression-based stemness scores (EREG-EXPss) (Figure 2F) revealed that FOXD3-AS1 was positively correlated with each stem cell index of GBM and LGG.

### Effects of FOXD3-AS1 on genes associated with RNA modification (m1A, m5C, and m6A)

**FIGURE 3:**
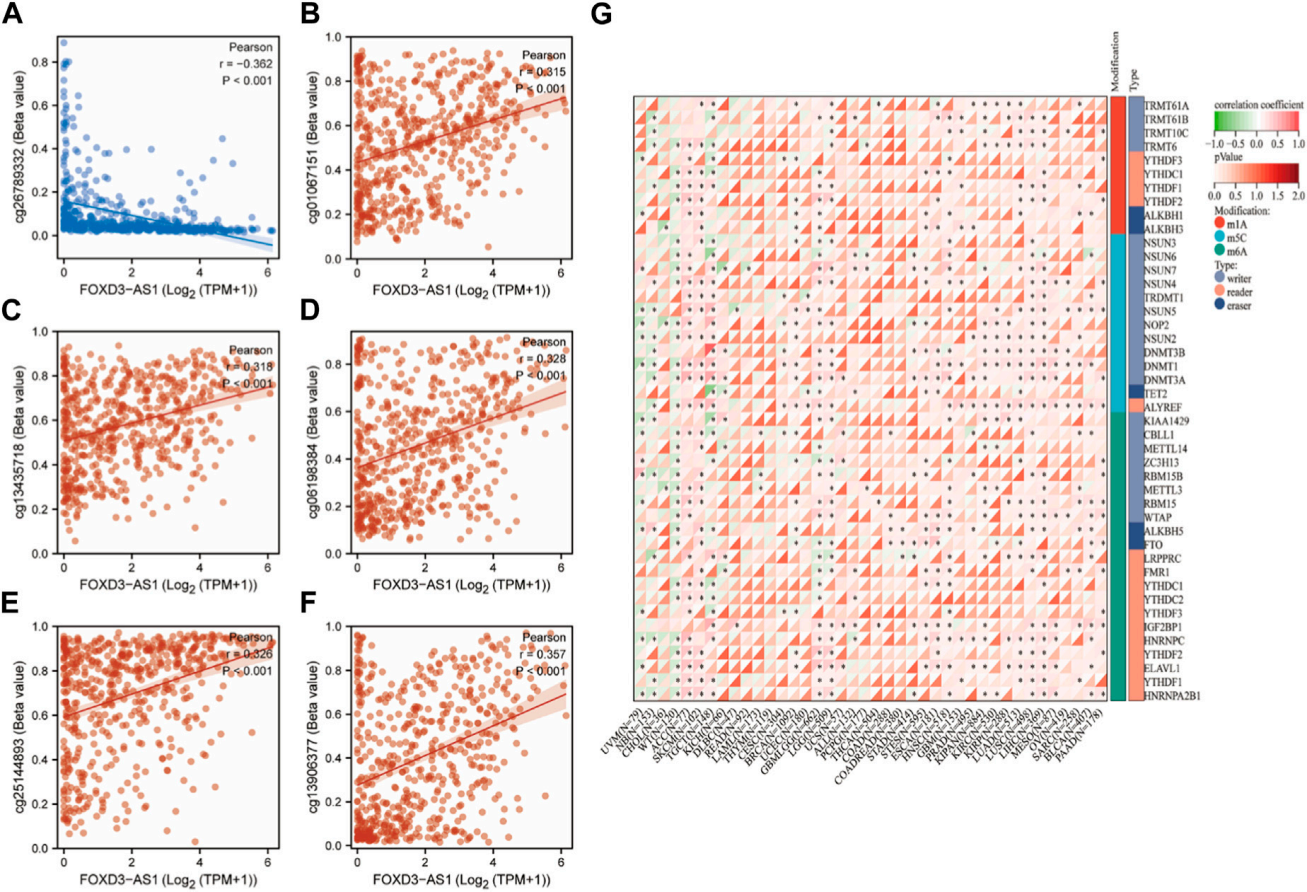
RNA modification correlation analysis of FOXD3-AS1. **(A–F)** FOXD3-AS1 in TCGA-GBMLGG is positively correlated with cg26789332 (r = − 0.362, *p* < 0.001), cg01067151 (r = 0.315, *p* < 0.001), cg13435718 (r = 0.318, *p* < 0.001), cg06198384 (r = 0.328, *p* < 0.001), cg25144893 (r = 0.326, *p* < 0.001) and cg13906377 (r = 0.357, *p* < 0.001) respectively; **(G)** Scatter diagram of FOXD3-AS1’s correlation with RNA modification related genes (m1A, m5C, m6A) in pan cancer.

The correlation between FOXD3-AS1 and cg26789332 (r = −0.362,*p* < 0.001), cg01067151 (r = 0.315, *p* < 0.001), cg13435718 (r = 0.318, *p* < 0.001), cg06198384 (r = 0.328, *p* < 0.001), cg25144893 (r = 0.326, *p* < 0.001), and cg13906377 (r = 0.357, *p* < 0.001) (Figure 3A) were calculated. Subsequently, the effect of FOXD3-AS1 on RNA modification-related genes (m1A, m5C, and m6A) in pan-cancer was investigated (Figure 3G), and the majority of them demonstrated a positive correlation in GBM and LGG.

### Differential expression and prognosis analysis of FOXD3-AS1 using the GEPIA database

**FIGURE 4:**
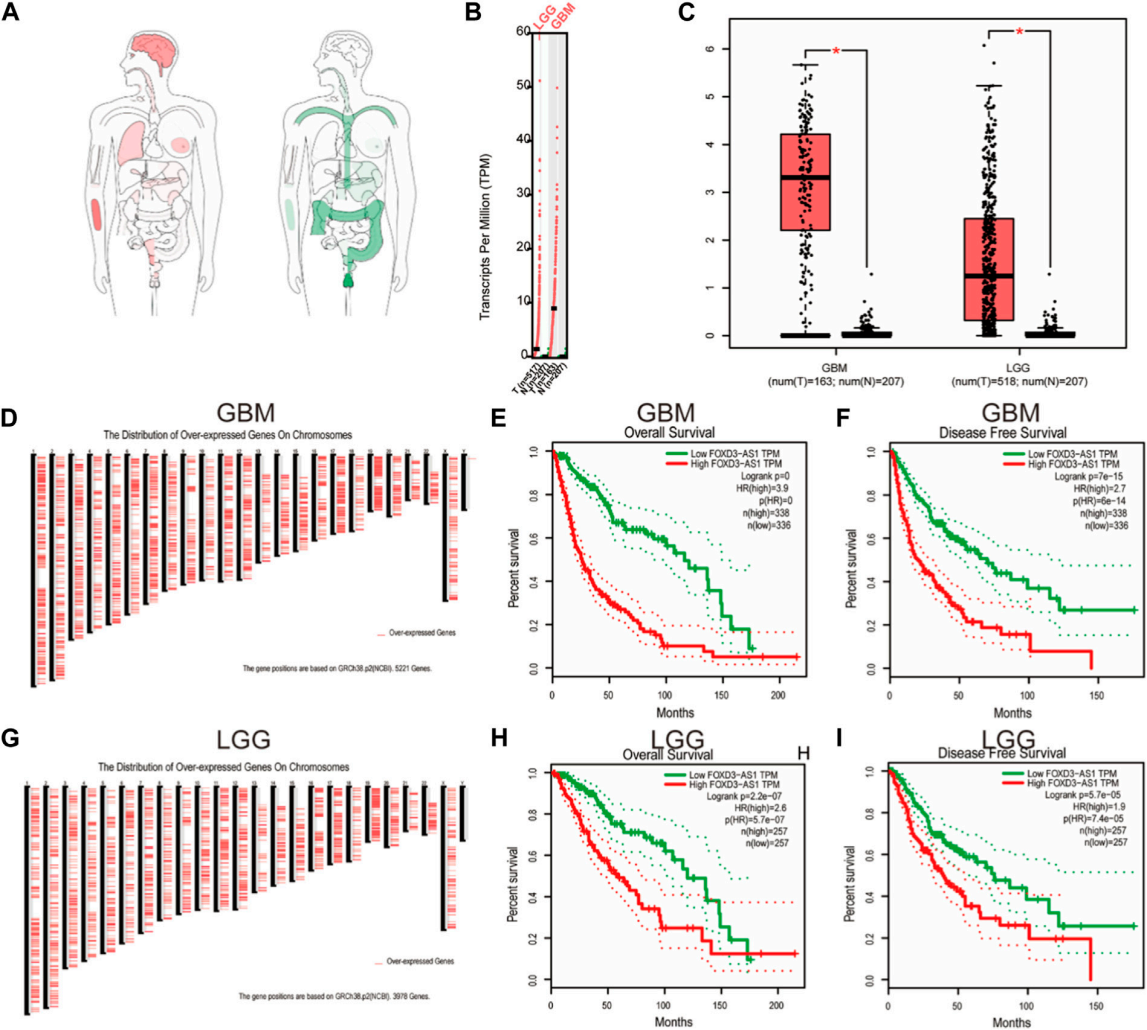
Differential expression of FOXD3-AS1 in GBM and LGG. **(A)** Interactive Bodymap shows the median expression of tumor tissue and normal tissue; **(B)** FOXD3-AS1 expression in GBM and LGG; **(C)** Differential expression FOXD3-AS1 of paired samples in TCGA database; **(D)** NCBI database is based on the location of GRCh38.p2 on chromosomes of 5221 overexpression genes in GBM; **(E, F)** GEPIA was used to analyze the survival curves of FOXD3-AS1 in GBM samples; **(G)** NCBI database is based on the location of GRCh38.p2 on chromosomes of 3978 overexpression genes in LGG; **(H, I)** GEPIA was used to analyze the levels of survival curves of FOXD3-AS1 expressed in GBM samples.

First, we investigated the overall expression difference of FOXD3-AS1 in tumors and normal tissues at different locations (Figure 4A) and varying expression levels (Figure 4B) using the GEPIA database and normal tissue samples from the GTEx database. Simultaneously, a box diagram was used to show the difference in FOXD3-AS1 expression between GBM and LGG samples (Figure 4C). Based on this, the positions of GRCh38.p2 (NCBI) on the chromosomes 5221 and 3978 overexpressing genes in GBM and LGG are displayed, respectively (Figures 4D,G). Subsequently, the survival and prognosis differences of FOXD3-AS1 in GBM and LGG are shown, respectively. The KM survival curve revealed that patients with high FOXD3-AS1 expression had poor OS and DSS (Figures 4E,F,H,I).

### Stratified analysis of FOXD3-AS1 expression differences based on clinical variables

**FIGURE 5:**
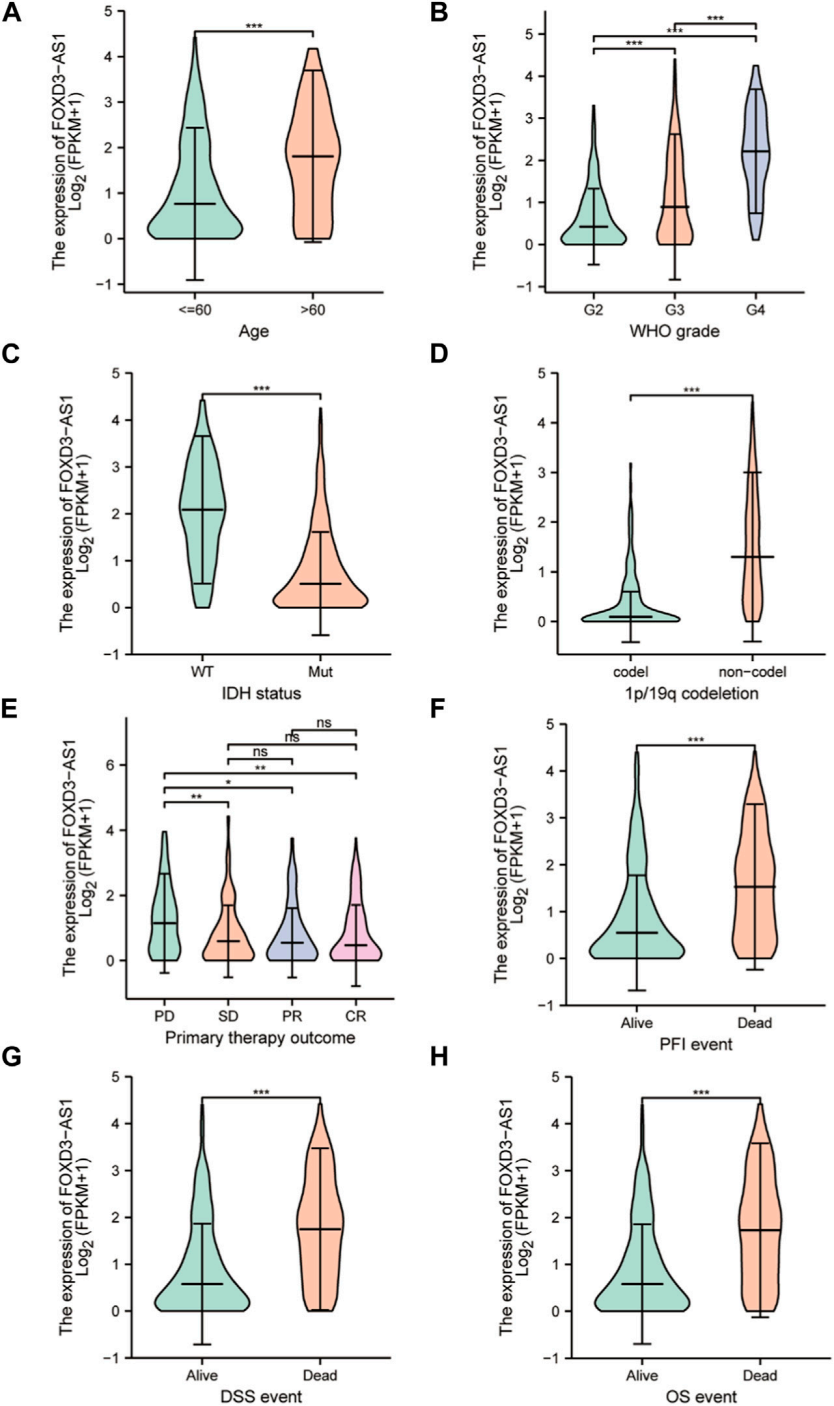
Determining the correlation between FOXD3-AS1 in TCGA and subgroups of GBM&LGG clinical variables. The box diagram shows the difference of FOXD3-AS1 expression among the subgroups of clinical variables of GBM and LGG, including **(A)** age, **(B)** WHO grade, **(C)** IDH status, **(D)** 1p/19q coding, **(E, F)** Primary therapy outcome, and the difference of expression among PFI **(G)**, DSS **(H)**, and OS survival states.

The differences in the subgroup of clinical variables were further analyzed. Differential expression stratification analysis revealed that the expression of FOXD3-AS1 was significantly different in age >60 (Figure 5A), G3 and G4 subgroup of the WHO graded (Figure 5B), the WT type in IDH status (Figure 5C), non-codel of the 1p/19q coded (Figure 5D), and the PD subgroup with initial treatment outcome (Figure 5E) were significantly higher. Similarly, FOXD3-AS1 was significantly overexpressed in PFI, DSS, and OS subgroups with poor prognosis (Figures 5F–H).

### Nomogram construction based on the clinically variable prognosis of GBM and LGG

**FIGURE 6:**
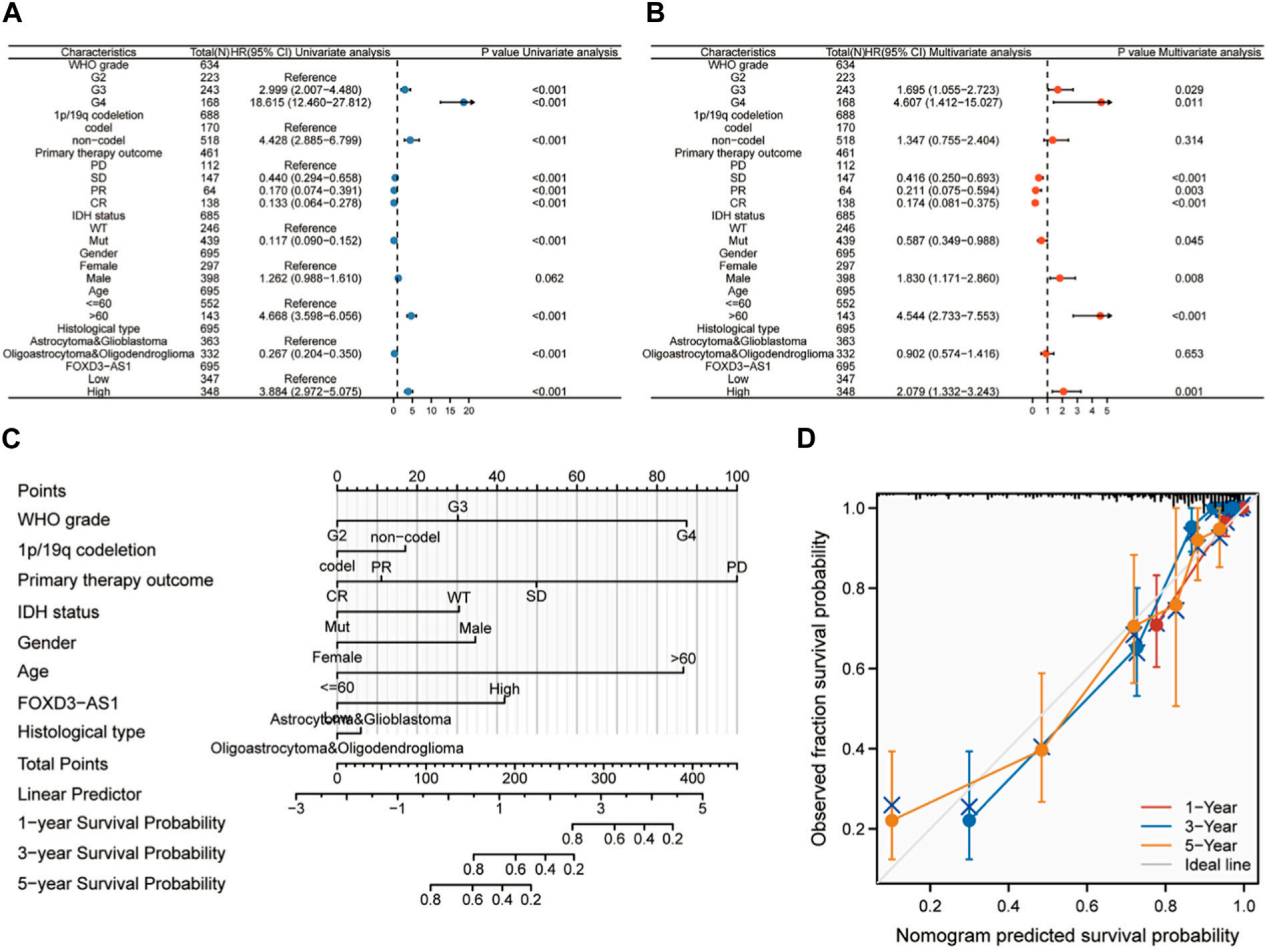
Cox regression analysis and nomogram construction based on FOXD3-AS1. **(A, B)** Univariate and multivariate Cox regression analysis based on FOXD3-AS1l combined with clinicopathological factors; **(C, D)** Prognostic nomogram and calibration curve of comprehensive clinicopathological factors in FOXD3-AS1 combined with GBM and LGG patients.

Univariate and multivariate Cox regression analyses were used based on the median expression value to investigate the independent prognostic value of FOXD3-AS1 in glioma. FOXD3-AS1 was identified as a risk factor (Figure 6A). Multivariate Cox regression analysis revealed that FOXD3-AS1, FIGO staging, and tumor residue were all independently related to OS, implying that FOXD3-AS1 may be an independent prognostic factor for EC patients (Figure 6B). G3 and G4 subgroups in the WHO grade, subgroups with an age >60, and men are high-risk factors for a poor prognosis in GBM and LGG patients, whereas SD, PR, and CR subtypes of primary therapy outcome and the Mut subtype of IDH status are protective factors. Simultaneously, the significant factors in the Cox analysis were combined to build a visual prognosis model (Figure 6C). The corresponding correction curve shows that the model has a good predictive value (Figure 6D; Supplementary Table S2).

**FIGURE 7:**
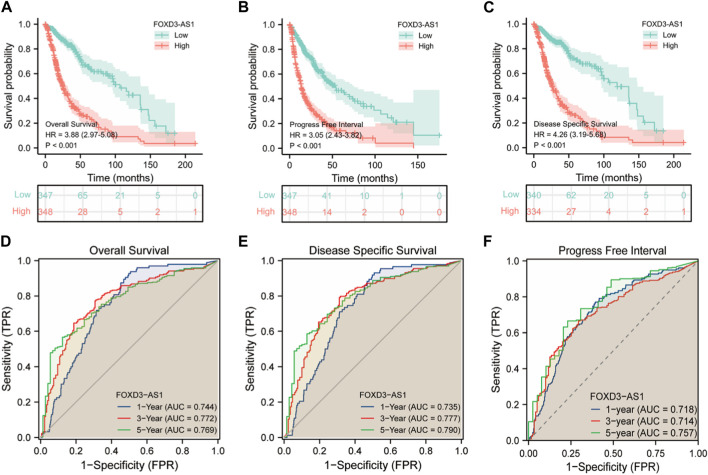
Correlation analysis of prognosis and survival of FOXD3-AS1. **(A–C)** were OS, DSS and PFI prognosis K-M survival curves between FOXD3-AS1 high and low expression groups; **(D–F)** FOXD3-AS1 is based on the ROC curve of OS, DSS and PFI 1 -, 3 -, 5-year survival status.

The results of the prognostic analysis were validated using the discriminative power of survival analysis and the ROC curve. First, the survival difference between high and low FOXD3-AS1 expression groups of OS, DSS, and PFI (Figures 7A–C). Subsequently, the predictive value of FOXD3-AS1 for 1, 3, and 5-year survival status of GBM and LGG patients based on OS was measured. The findings revealed that in the ROC prognostic analysis of 1-year survival of GBM and LGG patients by FOXD3-AS1, AUC = 0.744, AUC = 0.772 for 3-year, and AUC = 0.769 for 5-year (Figure 7D). It shows that FOXD3-AS1 has a better prognostic value in predicting survival in GBM and LGG patients. Furthermore, based on DSS and PFI survival time-dependent ROC analysis, FOXD3-AS1 expression can predict DSS [1-year (AUC = 0.735), 3-year (AUC = 0.777), and 5-year (AUC = 0.790)] and PFI [1-year (AUC = 0.735), 3-year (AUC = 0.777), and 5-year (AUC = 0.790)] in GBM and LGG patients (Figures 7E,F).

### Relationship between FOXD3-AS1 expression and prognosis

**FIGURE 8:**
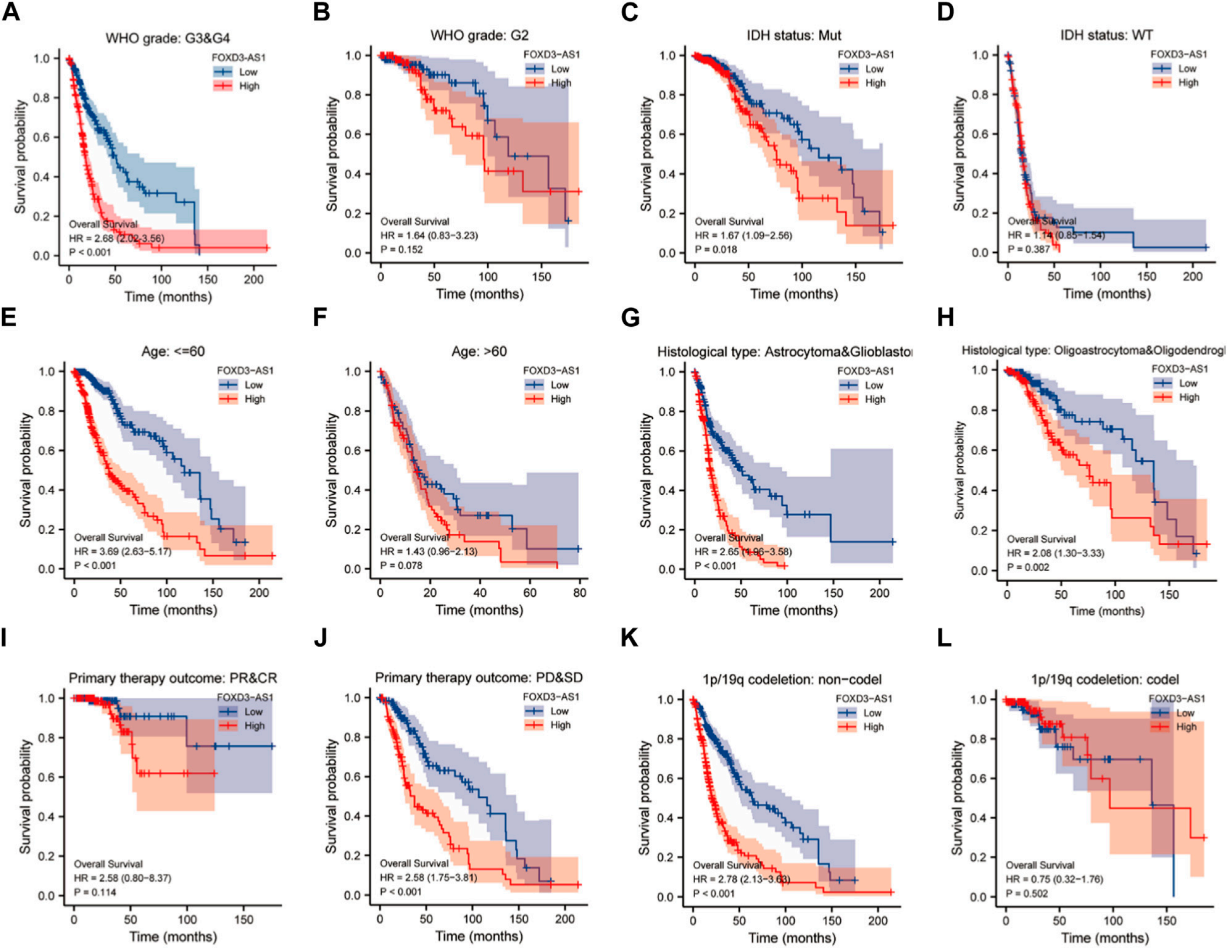
Prognostic value of KIF4A in EC patients. Kaplan-Meier stratified survival analysis of clinical adverse subgroups, respectively **(A, B)** WHO grade, **(C, D)** IDH status, **(E, F)** Age, **(G, H)** Histological type, **(I, J)** Primary therapy outcome **(K, L)** 1p/19q codeletion.

According to the survival analysis, high FOXD3-AS1 expression in GBM and LGG patients indicates a poor prognosis in each clinical variable subgroup of TCGA database. Therefore, it can be determined which subgroups of clinical variables with varying FOXD3-AS1 expression are most predictive of a poor prognosis. We stratified the analysis across multiple groups of pathological subtypes and found that high FOXD3-AS1 expression in most pathological types with poor prognoses for GBM and LGG indicates a significantly worse prognosis. Although the patients in the G2 subgroup of WHO grade pathological variables were not statistically significant, the prognosis of high FOXD3-AS1 expression in the G3 and G4 subgroups was poor [HR = 2.68 (2.02–3.56), *p* < 0.001] (Figures 8A,B). There was no statistical significance in WT patients with IDH, whereas the high expression of FOXD3-AS1 in the Mut patient population suggested a poor prognosis [HR = 1.67 (1.09–2.56), *p* = 0.018] (Figures 8C,D). The subgroup of patients with age ≤60 years and high FOXD3-AS1 had a poor prognosis [HR = 3.69 (2.63–5.17), *p* < 0.001], whereas the effect of FOXD3-AS1 expression in the subgroup of patients with age >60 years was not significant (Figures 8E,F). FOXD3-AS1 is a significant high-risk factor for poor prognosis of GBM and LGG in the historical type of astrocytoma and glioblastoma [HR = 2.65 (1.96–3.58), *p* < 0.001] and oligoastrocytoma and oligodendroglioma [R = 2.08 (1.30–3.33), *p* = 0.002] (Figures 8G,H). FOXD3-AS1 is not sensitive to prognosis prediction in PR and CR subgroup patients, but poor prognosis in the PD and SD populations is a risk factor [HR = 2.58 (1.75–3.81), *p* < 0.001] (Figures 8I,J). The worse prognosis of 1p/19q codec patients with non-codec was significantly related to the high expression of FOXD3-AS1 [HR = 2.78 (2.13–3.63) *p* < 0.001], while the correlation between 1p/19q codec patients and the high expression of FOXD3-AS1 was not strong (Figures 8K,L; Supplementary Table S3).

### Molecular network construction

**FIGURE 9:**
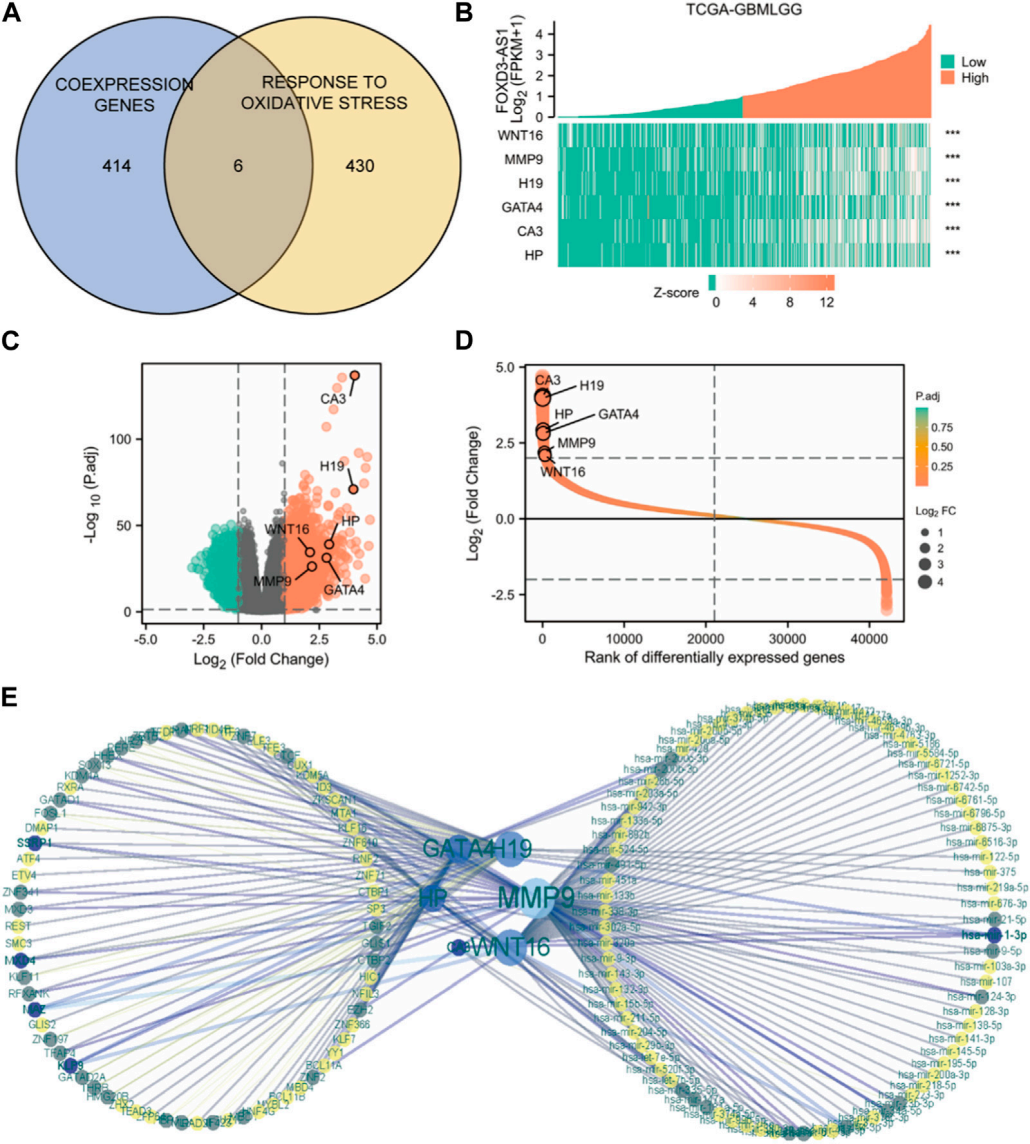
Molecular network construction. **(A)** Venn diagram of intersection of closely related co-expressed genes of FOXD3-AS1 and genes related to the molecular mechanism pathway of oxidative stress; **(B–D)** shows the differential expression heatmap **(B)**, Volcano map **(C)** and molecular differential expression fold ordering map **(D)** of FOXD3-AS1’s key oxidative stress related co-expressed genes among their expression groups, respectively; **(E)** miRNA or TF regulatory gene predictive molecular network targeting six oxidative stress co-expressed genes closely related to FOXD3-AS1.

The intersection of FOXD3-AS1 co-expressed genes with oxidative stress-related genes using Venn diagrams yielded six oxidative stress co-expressed genes closely associated with FOXD3-AS1 (Figure 9A). Figure 9B shows the differential expression heatmap of the key oxidative stress-related co-expressed genes of FOXD3-AS1 among its expression groups. Volcano plots (Figure 9C) and molecular differential expression fold sorting plots (Figure 9D) show that FOXD3-AS1 is significantly different from the key oxidative stress co-expression genes associated with it. To further understand the interaction of six oxidative stress co-expressed genes that are closely related to FOXD3-AS1 at the post-transcriptional level, we predicted the miRNA or transcription factor (TF) regulatory genes that they targeted. Following network construction, a total of 206 TF regulatory genes and 589 miRNAs were obtained (Figure 9E).

### Investigation of the mechanism underlying FOXD3-AS1

**FIGURE 10:**
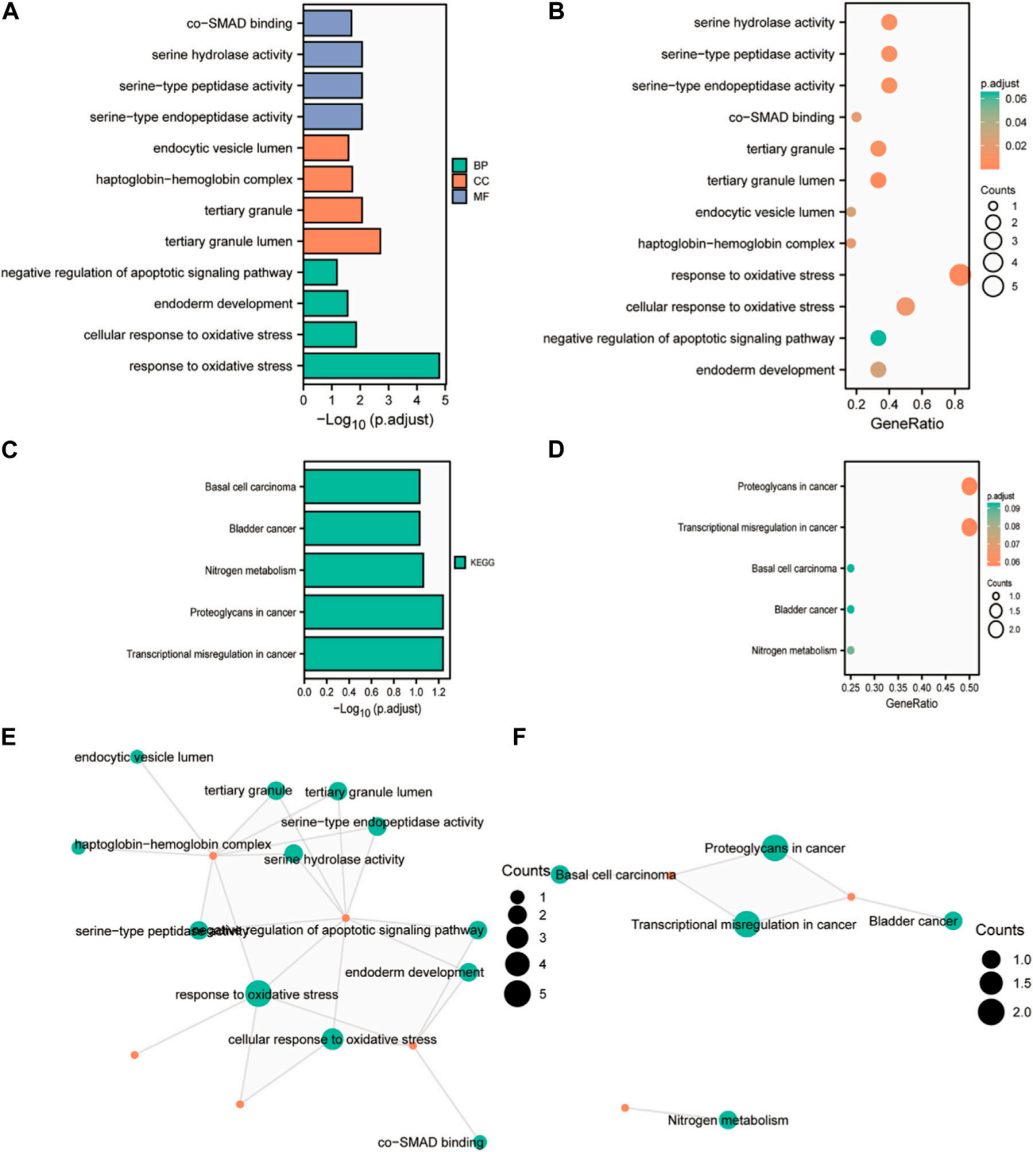
GO/KEGG enrichment analysis of closely related oxidative stress co-expressed genes. **(A, B)** Bar chart and bubble chart of GO enrichment analysis; **(C, D)** KEGG enrichment analysis bar chart and bubble chart; **(E, F)** chordal diagram of GO and KEGG enrichment analysis.

Because oxidative stress in nerve cells has been linked to malignant phenotypes such as tumor transformation, cell cycle dysregulation, and angiogenesis, GO and KEGG enrichment analyses were conducted for the six closely related oxidative stress co-expressed genes of FOXD3-AS1 to elucidate the potential mechanism of FOXD3-AS1 affecting glioma pathogenesis and prognosis. GO enrichment analysis revealed that biological process (BP) was enriched in response to oxidative stress, cellular response to oxidative stress, endoderm development, negative regulation of the apoptotic signaling pathway, molecular function (MF) was enriched in serine-type endopeptidase activity, serine-type peptidase activity, serine hydrolase activity, and co-SMAD binding, and cellular component (CC) enrichment entries are tertiary granule lumen, tertiary granule, haptoglobin–hemoglobin complex, and endocytic vesicle lumen (Figures 10A,B,E; Supplementary Table S3). KEGG pathways were enriched in transcriptional dysregulation in cancer, proteoglycans in cancer, nitrogen metabolism, bladder cancer, basal cell carcinoma, and other tumor-related pathways (Figures 10C–F; Supplementary Tables S4, S5).

### Gene set enrichment analysis

**FIGURE 11:**
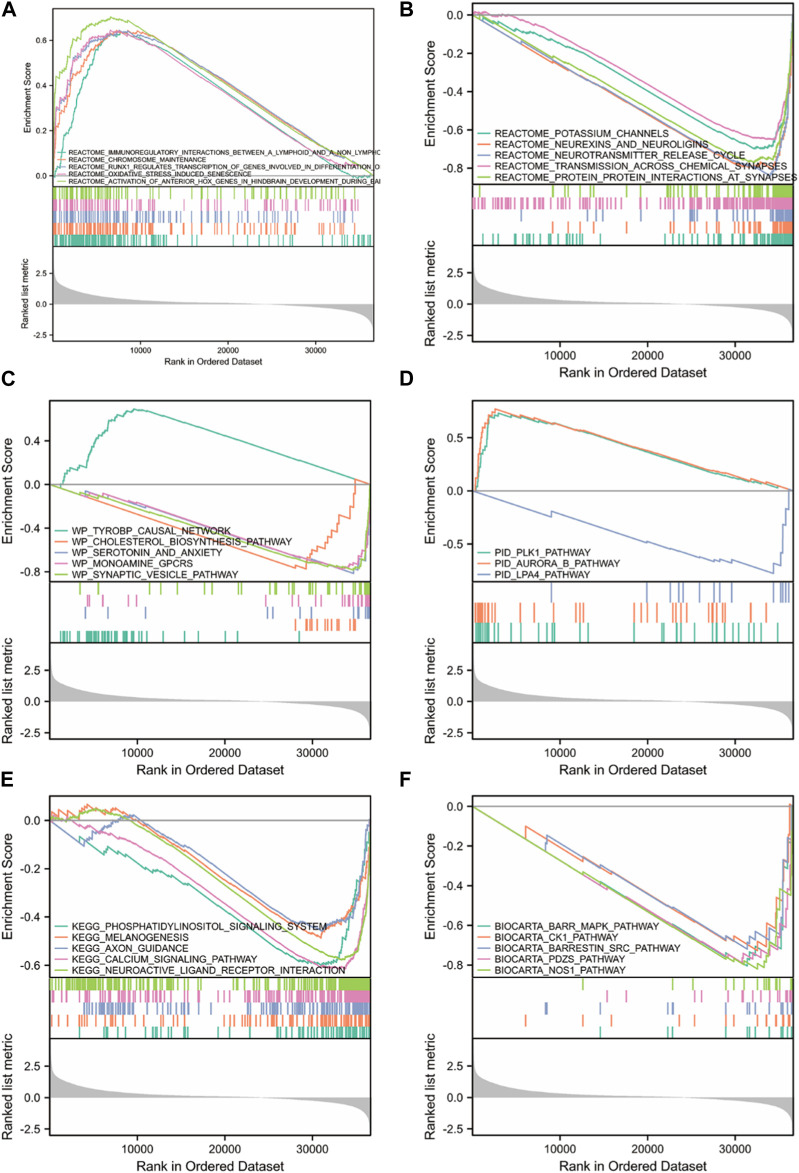
GSEA enrichment analysis between high and low FOXD3-AS1 expression groups. Significant enrichment entries were included in the REACTOME **(A, B)**, WP **(C)**, PID **(D)**, KEGG **(E)**, and BIOCARTA **(F)** databases, respectively.

To understand the biological molecular mechanism underlying FOXD3-AS1, we performed GSEA between high and low FOXD3-AS1 expression groups, with c2.all.v7.2. symbols.gmt as the background set (Figure 11; Supplementary Table S5). The attached GESA enrichment heatmap shows the significantly enriched entries for samples with high and low FOXD3-AS1 expression levels. Figure 11 shows enriched and statistically significant results from the REACTOME, WP, PID, KEGG, and BIOCARTA databases.

Multiple biological processes related to oxidative stress and molecular pathways related to immunotherapy response were significantly enriched in the REACTOME database, as well as CD22/BCR REGULATION, DNA methylation, CALCIUM MOBILIZATION, rRNA expression, and mobilization. prc2 methylation histone, IL10 synthesis transcription regulation RNA polymerase I promoter escape formation process, etc. (Figures 11A,B). The PID database was enriched in PLK1, AURORA B, and LPA4 (Figure 11D). The WP database was enriched in postsynaptic signaling pathway destruction, neural conduction calcium signal and its regulation, synaptic proteins of cholesterol metabolism, and synapses related to neural dysfunction (Figure 11C). The KEGG database was enriched in lupus erythematosus, long-term depression in amyotrophic lateral sclerosis, myocardial contraction, the phosphatidylinositol signaling system, melanogenesis, axonal guidance, the calcium signaling pathway, and neuroactive ligand-receptor interaction pathways (Figure 11E).

### Estimation of tumor purity in glioma based on FOXD3-AS1 expression

Based on the findings of this study, we hypothesized that FOXD3-AS1 may affect the prognosis by regulating the immune microenvironment and tumor stemness in GBM and LGG, as well as having the potential to affect the efficacy of immunotherapy.

### Potential prognostic value of FOXD3-AS1 in the immune system invasion

**FIGURE 12:**
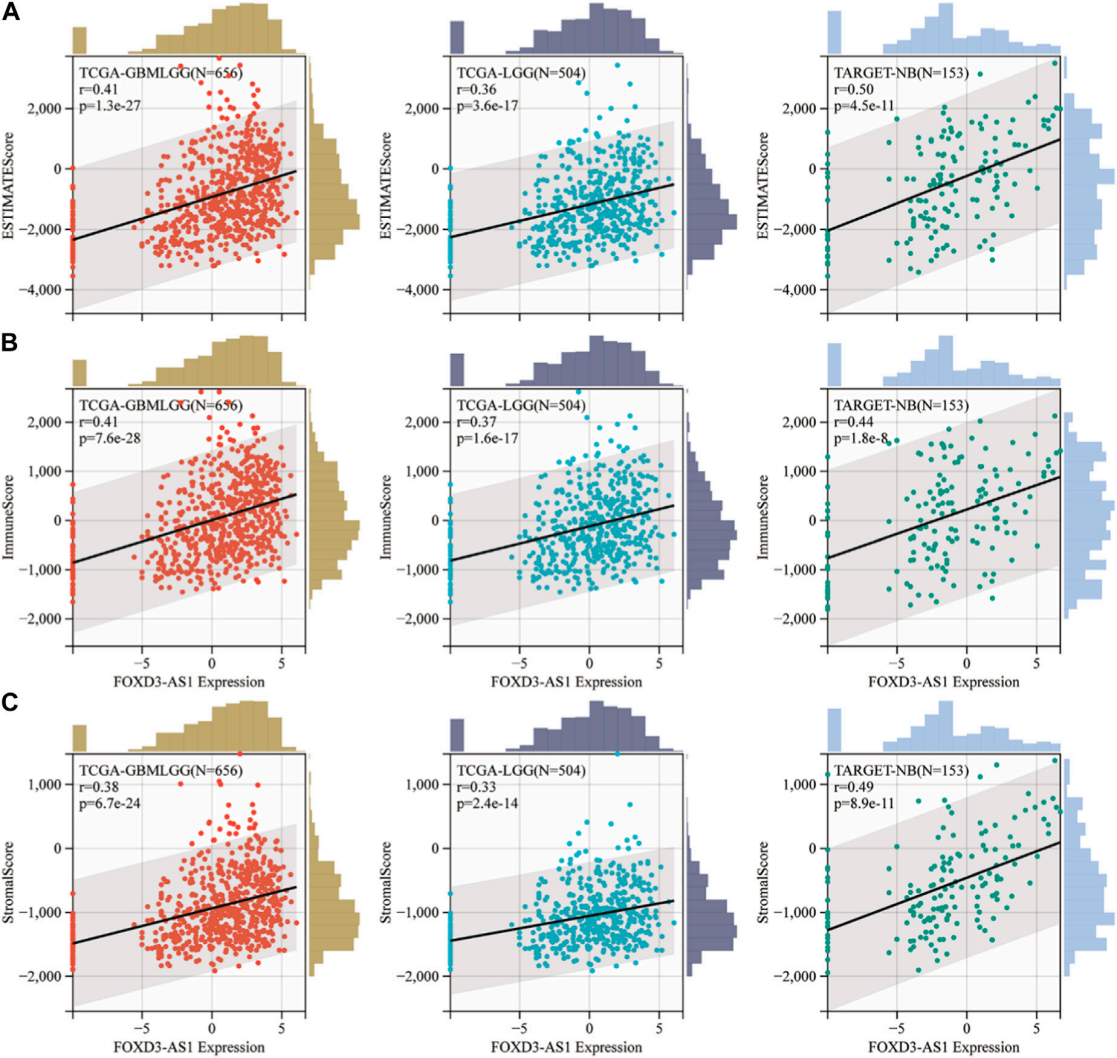
ESTIMATE algorithm was used to analyze the immune infiltration status of FOXD3-AS1 in tumors. Scatter plots of the first 3 significant correlations between FOXD3-AS1 expression and ESTIMATEScore **(A)**, ImmuneScore **(B)** and StromalScore **(C)**.

The ESTIMATE algorithm was used to study the immune invasion state of the tumor. The ESTIMATE algorithm uses gene expression data to calculate the matrix score, immune score, and evaluation score and infers tumor purity based on these values. The scatter plots show the first three significant correlations between FOXD3-AS1 expression and ESTIMATEScore (Figure 12A), ImmunoScore (Figure 12B), and StromalScore (Figure 12C). The results are relatively consistent.

### Analysis of the prevalence of FOXD3-AS1 in immune cell infiltration in tumors

**FIGURE 13:**
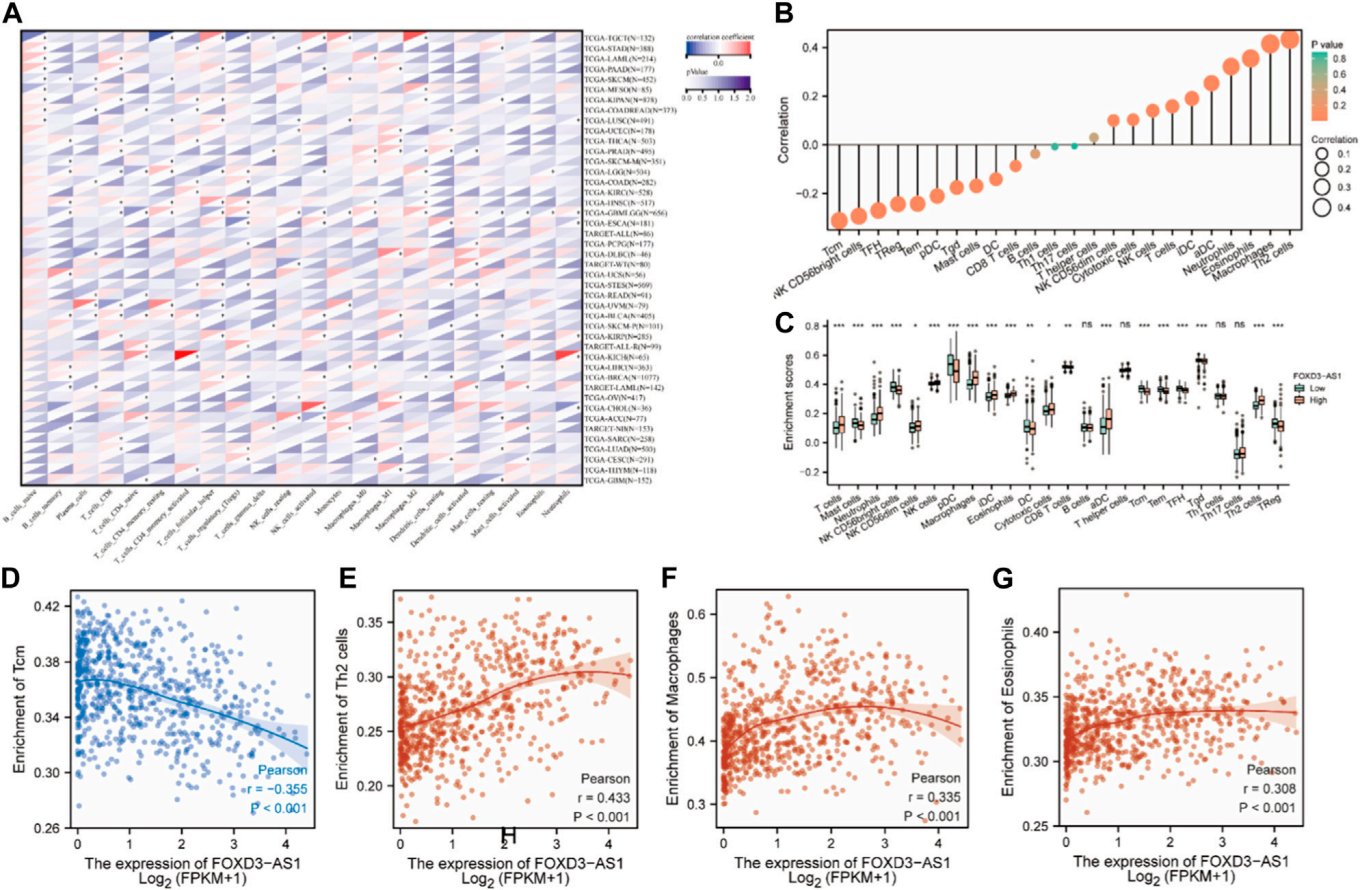
Abundance analysis of immune checkpoints and immune cell infiltration. **(A)** Heat map of FOXD3-AS1 correlation with 22 different immune cell types in pan-cancer; **(B)** The lollipop chart showing different degrees of correlation between FOXD3-AS1 and immune cells; **(C)** Boxplots of immune cell infiltration differences between high and low FOXD3-AS1 expression groups; **(D–G)** Scatter plots of correlation analysis between FOXD3-AS1 expression and Enrichment of Tcm **(D)**, and nrichment of Th2 cells **(E)**, respectively. Enrichment of Macrophage **(F)**, Enrichment of Eosinophils **(G)**.

The correlation heatmap with 22 different immune cell types in pan-cancer reveals that FOXD3-AS1 has a negative correlation trend with various immune cells in the majority of cancers in the US military (Figure 13A). The lollipop diagram (Figure 13B) of FOXD3-AS1 related to immune cells in different degrees and the box graph (Figure 13C) of the differences in immune cell infiltration between high and low expression groups show that FOXD3-AS1 is significantly correlated with T cells, mast cells, neural cells, CD56^bright^ natural killer (NK) cells, CD56^dim^ NK cells, NK cells, pDCs, iDCs, aDCs, DCs, macrophages, eosinophils, cytotoxic cells, CD8 T cells, Th cells, Tcm cells, Tem cells, TFH cells, Tgd cells, and Treg cells.

**FIGURE 14:**
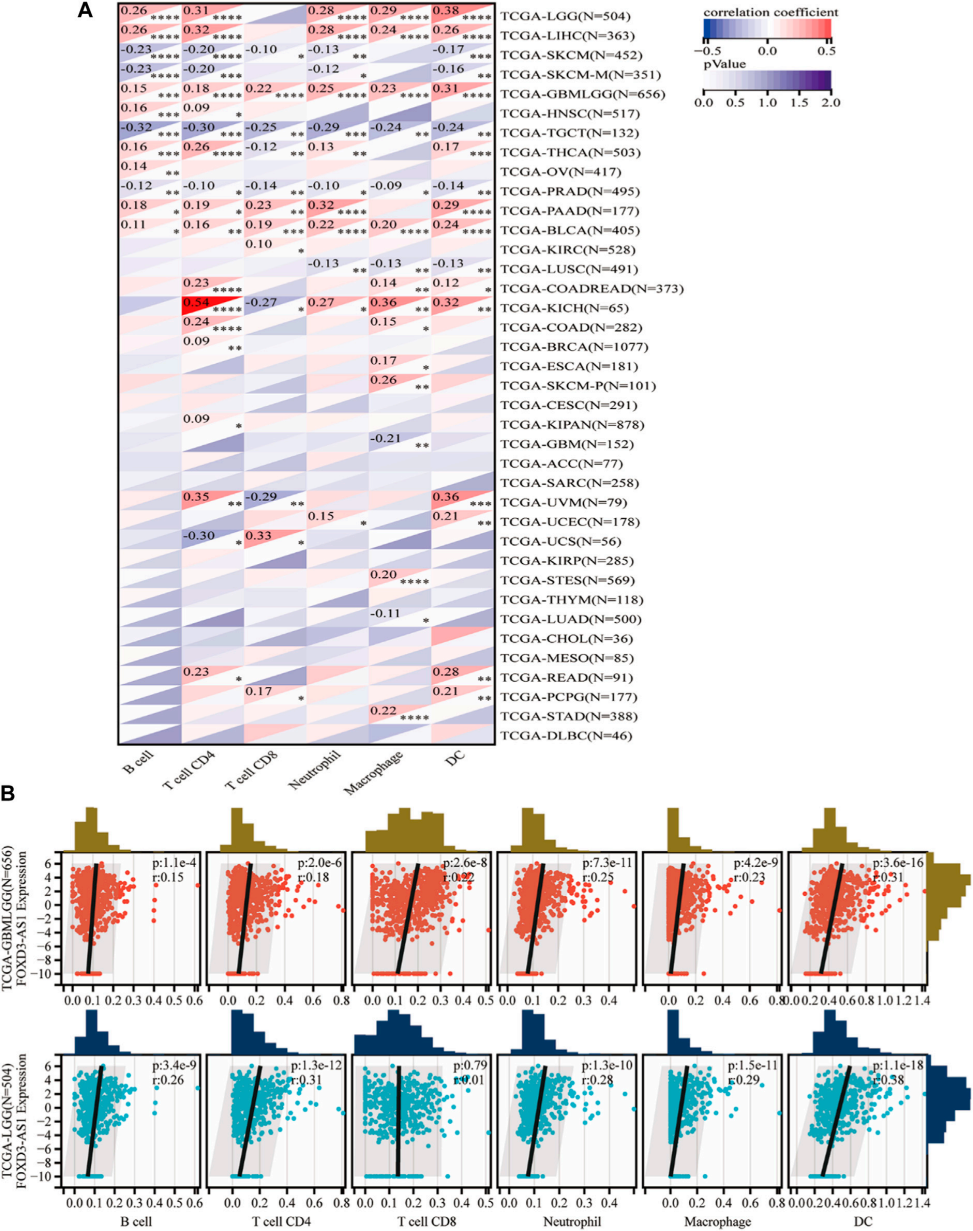
Timer database evaluation and verify. **(A)** The correlation between FOXD3-AS1 and B cell, T cell CD4, T cell CD8, Neutrophil, Macrophage, and DC infiltration scores in pan cancer; **(B)** Scatter plots of significant correlations between FOXD3-AS1 and B cell, T cell CD4, T cell CD8, Neutrophil, Macrophage, and DC infiltration scores in GBM and LGG assessed by Timer.

The scatter plot of the correlation analysis between FOXD3-AS1 expression and immune cells revealed that FOXD3-AS1 was negatively correlated with the enrichment of Tcm cells (r = −0.355, *p* < 0.001) and positively correlated with the enrichment of Th2 cells (r = 0.433, *p* < 0.001), macrophages (r = 0.335, *p* < 0.001), and eosinophils (r = 0.308, *p* < 0.001) (Figures 13D–G; Supplementary Table S7). Although FOXD3-AS1 is significantly and positively correlated with the matrix score, immune score, and evaluation score in glioma, the standardized matrix score is secondary, indicating that samples with low immune invasion have high FOXD3-AS1 expression. This is consistent with patients with high FOXD3-AS1 expression having a poor prognosis. The TIMER database was used to evaluate and validate the correlation between FOXD3-AS1 expression and immune cell infiltration scores in pan-cancer (Figure 14A). The positive correlation between the immune cell infiltration fraction and FOXD3-AS1 in GBM and LGG is shown in a scatter plot (Figure 14B).

### FOXD3-AS1 promotes glioma cell migration and invasion *in vitro*

**FIGURE 15:**
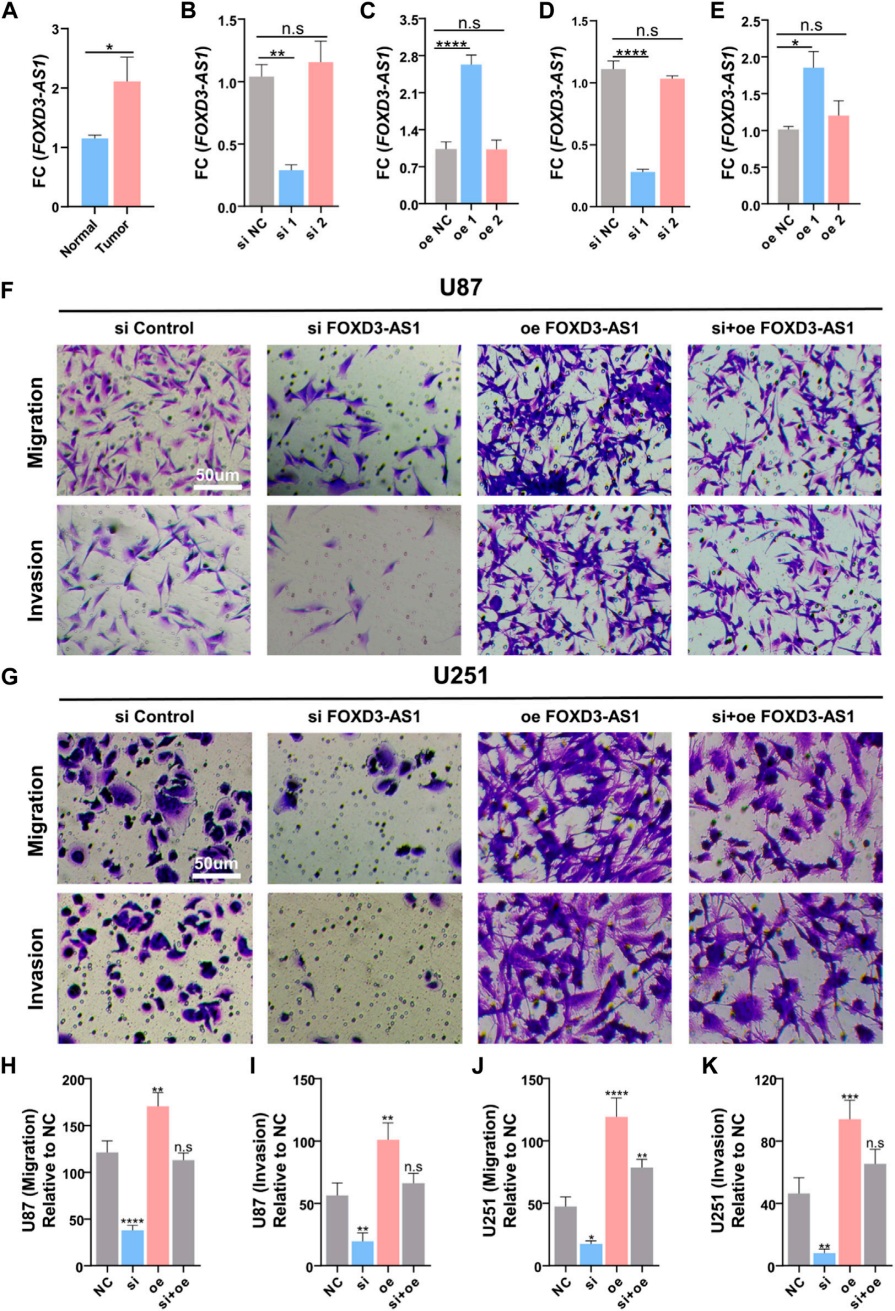
FOXD3-AS1 promotes glioma cell migratory and invasive capacity *in vitro*. **(A)** Results of RT-PCR assay on tumor tissues and paraneoplastic tissues, FOXD3-AS1 was elevated in tumor tissues. **(B–E)** Detection of transfection efficiency of si-FOXD3-AS1 and FOXD3-AS1-overexpression in U-87 cell line and U-251 cell line using RT-PCR, and standardization of the results **(F, G)** Transwell assay images of migration and invasion in the negative control, FOXD3-AS1 knockout groups, FOXD3-AS1 overexpression and FOXD3-AS1 rescue groups. **(H–K)** Quantitative analysis of migrating and invading glioma cells. n.s *P* › 0.05, **p ≤ 0.01, ***p ≤ 0.001, ****p ≤ 0.001.

FOXD3-AS1 was tested for its ability to promote glioma cell migration and invasion *in vitro*. RT–PCR detected a difference in FOXD3-AS1 expression between tumor and paraneoplastic tissue, indicating that the expression in tumor tissue was increased (Figure 15A). Subsequently, the transfection efficiency of si-FOXD3-AS1 and oe-FOXD3-AS1 in U-87 and U-251 cell lines was determined using RT–PCR, and the results were standardized (Figures 15B–E). Transwell assay images showed the migration and invasion of negative control group, FOXD3-AS1 knockdown group, FOXD3-AS1 overexpression group and FOXD3-AS1 rescue group, as well as quantitative analysis of glioma cell migration and invasion, indicating that interference with FOXD3-AS1 expression could promote the migration and invasion of glioma cells *in vitro* to some extent, and we found that the migration and invasion of U87 and U251 cell lines were enhanced after overexpression of FOXD3-AS1 (Figures 15F–K).

### FOXD3-AS1 is associated with oxidative stress in U87 and U251 cell lines

**FIGURE 16:**
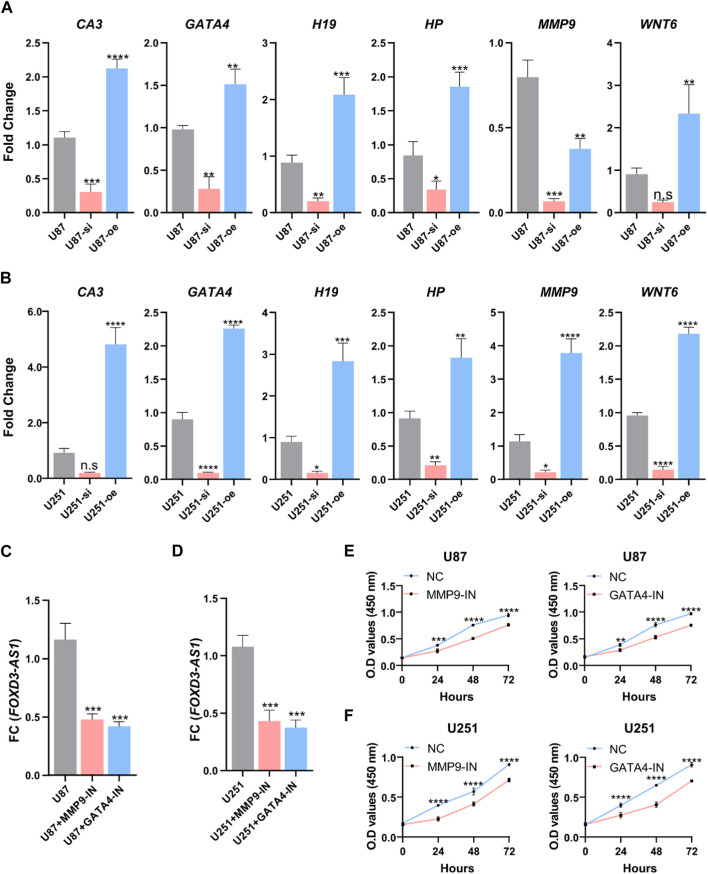
FOXD3-AS1 is associated with oxidative stress in U87 and U251 cell lines. **(A)** RT-PCR was performed to detect and relatively quantify the expression of *CA3*, *GATA4*, *H19*, *HP*, *MMP9*, and *WNT6* associated with oxidative stress in the U87 cell line. **(B)** RT-PCR was performed to detect and relatively quantify the expression of *CA3*, *GATA4*, *H19*, *HP*, *MMP9*, and *WNT6* associated with oxidative stress in the U251 cell line. **(C, D)** The expression of FOXD3-AS1 was detected and quantified by interfering with the oxidative stress pathway in U87 and U251 cell lines using inhibitors. **(E, F)** Cellular activity was detected and quantified by interfering with the oxidative stress pathway in U87 and U251 cell lines using inhibitors. n.s *P* › 0.05, **p ≤ 0.01, ***p ≤ 0.001, ****p ≤ 0.001.

Based on the results of the bioinformatics analysis, we selected *CA3*,*GATA4*,*H19*,*HP*, *MMP9*, and *WNT6*, which are the six most relevant makers of oxidative stress in this model, to do the validation in U87 and U251 cell lines, and the results showed that the expression of FOXD3-AS1 was associated with the imbalance of oxidative stress in glioma cell lines, after interference with the expression of FOXD3-AS1 in U87 and U251 FOXD3-AS1 expression significantly decreased the expression of oxidative stress makers, while overexpression of FOXD3-AS1 significantly increased the expression of oxidative stress makers (Figures 16A,B). Subsequently, we interfered with the expression of oxidative stress pathway in U87 as well as U251 cell lines using common MMP9 inhibitors as well as GATA4 inhibitors, and found a decrease in FOXD3-AS1 expression (Figures 16C,D). It was also found that the activity of the cells was reduced after interfering with the expression of oxidative stress pathway in U87 as well as U251 cell lines (Figures 16E,F).

## Discussion

Gliomas, which originate from glial precursor cells, are among the most difficult brain tumors to treat because of their rapid proliferation and high invasiveness. Drug therapy for glioma is hindered by the presence of the blood-brain barrier (BBB), which causes ineffective drug allocation and drug resistance. Although several FDA-approved multimodal therapies for glioblastoma are available, the majority of patients still have poor prognoses. With the discovery of new molecular mechanisms in recent years, targeted epigenetic therapy, immunotherapy, gene therapy, and vaccine and peptide therapy for glioma have emerged as innovative methods to increase the efficiency of anti-glioma treatment. Immunotherapy, in particular, is a hot potential direction in cancer treatment because of its ability to penetrate the BBB.

FOXD3-AS1 is an emerging potential target for tumor prediction and treatment, and many studies have reported on its potential application value. However, the utility of FOXD3-AS1 in glioma has not been reported. In this study, FOXD3-AS1 was thoroughly investigated in gliomas. The FOXD3-AS1 genetic map of TCGA-GBM and LGG revealed a strong association with clinicopathological variables. Several studies have confirmed the significant prognostic potential of FOXD3-AS1. Therefore, the biomarker identified in this work, FOXD3-AS1, can be used to assess treatment efficacy and survival outcomes in patients with glioma.

The differential expression of FOXD3-AS1 in GBM and LGG was analyzed in TCGA database. Different survival curves were obtained for GBM and LGG samples, indicating that the high expression of FOXD3-AS1 was associated with a poor prognosis and survival outcome. The prognostic value of FOXD3-AS1 in GBM and LGG patients was determined. The expression of FOXD3-AS1 differed significantly between clinical subgroups of GBM and LGG patients, with the high FOXD3-AS1 subgroup being significantly associated with a poor prognosis. The subsequent Cox regression analysis of FOXD3-AS1 as a high-risk factor for glioma affected prognostic outcomes independently. The survival analysis revealed that patients with a high level of FOXD3-AS1 had a poor prognosis. The differential expression of FOXD3-AS1 in various clinical subgroups as well as the predictive value of FOXD3-AS1 for each clinical subgroup was analyzed.

Based on these findings, we hypothesized that FOXD3-AS1 has the potential to alter the immune microenvironment and thereby affect prognosis. The expression of immune cell infiltration and immune-related signaling markers was compared between FOXD3-AS1 high- and low-expression subgroups. Correlation analysis indicates that there are significant differences in immune cell infiltration among immune cell subsets, which may also contribute to the advancement of glioma immunotherapy research.

To the best of our knowledge, this is the first report to investigate FOXD3-AS1 in glioma. FOXD3-AS1 has the potential to influence prognosis by regulating the GBM and LGG immune microenvironments. A stratified clinicopathological subgroup analysis revealed that a high level of FOXD3-AS1 is associated with a poor prognosis. This also indicates a link between FOXD3-AS1 and tumorigenesis and prognosis, which has potential application value.

FOXD3-AS1 was detected in tumor and paraneoplastic tissues using RT-qPCR. Transwell analysis also verified and confirmed the migration and invasion of the FOXD3-AS1 knockout group *in vitro* to a certain extent. We hope that this study will fill the FOXD3-AS1 gap in the field of glioma, provide valuable information, and lay the foundation for future clinical research.

Although these findings were validated in glioma cell assays, prospective clinical studies with larger sample sizes are urgently needed to evaluate our results. Second, we comprehensively explored the immunological role of FOXD3-AS1 in glioma; however, the mechanism between FOXD3-AS1 and the immune response is unclear. Detailed experimental studies should focus on *in vivo* studies to explore the potential mechanisms of FOXD3-AS1 regarding immune cell infiltration. Third, although this study used bioinformatics to analyze the correlation between FOXD3-AS1 and multiple TIICs and RT-qPCR experiments to preliminarily explore the changes of oxidative stress genes in FOXD3-AS1-overexpression and Inhibition cells, a certain amount of clinical specimens and co-culture experiments are needed to verify these results. In summary, FOXD3-AS1 may interact with TME components involved in various cancer, oxidative stress and immune-related pathways and play an important role in the progression of glioma. It is hoped that the findings will provide informative information for clinical application and medical decision making. Future large-scale prospective studies are needed to validate all results.

## Data Availability

The original contributions presented in the study are included in the article/Supplementary Material, further inquiries can be directed to the corresponding author.
