# Supplementary material for: Prognostic value and immune-infiltration pattern of FOXD3-AS1 in patients with glioma
Source: Front Pharmacol. 2023 Apr 4;14:1162309. doi: 10.3389/fphar.2023.1162309 (PMC10110859; doi:10.3389/fphar.2023.1162309)
Supplement: Supplementary file 1 [file Table5.pdf]

Supplementary Table 5. KEGG enrichment results of six oxidative stress co-expressed genes closely related to FOXD3-AS1 in the TCGA-GBMLGG cohort

Ontology	ID	Description	p.adjust	qvalue
KEGG	hsa05202	Transcriptional misregulation in cancer	0.058	0.043
KEGG	hsa05205	Proteoglycans in cancer	0.058	0.043
KEGG	hsa00910	Nitrogen metabolism	0.087	0.065
KEGG	hsa05219	Bladder cancer	0.093	0.070
KEGG	hsa05217	Basal cell carcinoma	0.093	0.070
