# Supplementary material for: Prognostic value and immune-infiltration pattern of FOXD3-AS1 in patients with glioma
Source: Front Pharmacol. 2023 Apr 4;14:1162309. doi: 10.3389/fphar.2023.1162309 (PMC10110859; doi:10.3389/fphar.2023.1162309)
Supplement: Supplementary file 2 [file Table4.pdf]

Supplementary Table 4. GO enrichment results of six oxidative stress co-expressed genes closely related to FOXD3-AS1 in the TCGA-GBMLGG cohort

Ontology	ID	Description	p.adjust	qvalue
BP	GO:0006979	response to oxidative stress	1.62e-05	7.07e-06
BP	GO:0034599	cellular response to oxidative stress	0.014	0.006
BP	GO:0007492	endoderm development	0.028	0.012
BP	GO:2001234	negative regulation of apoptotic signaling pathway	0.066	0.029
CC	GO:1904724	tertiary granule lumen	0.002	9.58e-04
CC	GO:0070820	tertiary granule	0.009	0.004
CC	GO:0031838	haptoglobin-hemoglobin complex	0.019	0.009
CC	GO:0071682	endocytic vesicle lumen	0.026	0.013
MF	GO:0004252	serine-type endopeptidase activity	0.009	0.003
MF	GO:0008236	serine-type peptidase activity	0.009	0.003
MF	GO:0017171	serine hydrolase activity	0.009	0.003
MF	GO:0070410	co-SMAD binding	0.020	0.006
