# Supplementary material for: Prognostic value and immune-infiltration pattern of FOXD3-AS1 in patients with glioma
Source: Front Pharmacol. 2023 Apr 4;14:1162309. doi: 10.3389/fphar.2023.1162309 (PMC10110859; doi:10.3389/fphar.2023.1162309)
Supplement: Supplementary file 3 [file Table1.pdf]

Supplementary Table 1. Baseline analysis of clinical information in TCGA-GBMLGG patients

Characteristic	Low expression of FOXD3-AS1	High expression of FOXD3-AS1	p
n	348	348	
WHO grade, n (%)			< 0.001
G2	166 (26.1%)	58 (9.1%)	
G3	123 (19.4%)	120 (18.9%)	
G4	25 (3.9%)	143 (22.5%)	
IDH status, n (%)			< 0.001
WT	42 (6.1%)	204 (29.7%)	
Mut	299 (43.6%)	141 (20.6%)	
Gender, n (%)			0.491
Female	144 (20.7%)	154 (22.1%)	
Male	204 (29.3%)	194 (27.9%)	
Primary therapy outcome, n (%)			< 0.001
PD	47 (10.2%)	65 (14.1%)	
SD	101 (21.9%)	46 (10%)	
PR	45 (9.7%)	19 (4.1%)	
CR	88 (19%)	51 (11%)	
1p/19q codeletion, n (%)			< 0.001
codel	147 (21.3%)	24 (3.5%)	

Characteristic	Low expression of FOXD3-AS1	High expression of FOXD3-AS1	p
non-codel	201 (29.2%)	317 (46%)	
Race, n (%)			0.701
Asian	5 (0.7%)	8 (1.2%)	
Black or African American	16 (2.3%)	17 (2.5%)	
White	319 (46.7%)	318 (46.6%)	
Histological type, n (%)			< 0.001
Astrocytoma	93 (13.4%)	102 (14.7%)	
Glioblastoma	25 (3.6%)	143 (20.5%)	
Oligoastrocytoma	72 (10.3%)	62 (8.9%)	
Oligodendroglioma	158 (22.7%)	41 (5.9%)	
Age, median (IQR)	40 (32, 51)	53 (38, 63)	< 0.001
