# Supplementary material for: Prognostic value and immune-infiltration pattern of FOXD3-AS1 in patients with glioma
Source: Front Pharmacol. 2023 Apr 4;14:1162309. doi: 10.3389/fphar.2023.1162309 (PMC10110859; doi:10.3389/fphar.2023.1162309)
Supplement: Supplementary file 4 [file Table2.pdf]

Supplementary Table 2. Logistic regression analysis between FOXD3-AS1 and GBM&LGG clinical variables in TCGA

Characteristics	Total(N)	Odds Ratio(OR)	P value
WHO grade (G3&G4 vs. G2)	635	5.086 (3.565-7.337)	<0.001
1p/19q codeletion (non-codel vs. codel)	689	9.660 (6.168-15.728)	<0.001
Primary therapy outcome (PR&CR vs. PD&SD)	462	0.702 (0.479-1.024)	0.068
IDH status (Mut vs. WT)	686	0.097 (0.065-0.142)	<0.001
Histological type (Oligoastrocytoma&Oligodendroglioma vs. Astrocytoma&Glioblastoma)	696	0.216 (0.156-0.296)	<0.001
Age (>60 vs. <=60)	696	3.105 (2.097-4.669)	<0.001
Gender (Male vs. Female)	696	0.889 (0.658-1.201)	0.444
