# Supplementary material for: Prognostic value and immune-infiltration pattern of FOXD3-AS1 in patients with glioma
Source: Front Pharmacol. 2023 Apr 4;14:1162309. doi: 10.3389/fphar.2023.1162309 (PMC10110859; doi:10.3389/fphar.2023.1162309)
Supplement: Supplementary file 6 [file Table6.pdf]

**Supplementary Table 6. GSEA results of high and low expression isoforms of FOXD3-AS1 in the TCGA-GBMLGG cohort.**

| Description | ES | NES | adj.P | q |
|---|---|---|---|---|
| BIOCARTA_BARR_MAPK_PATHWAY | -0.80 | -2.09 | 0.04 | 0.03 |
| BIOCARTA_BARRESTIN_PATHWAY | -0.87 | -2.18 | 0.04 | 0.03 |
| BIOCARTA_BARRESTIN_SRC_PATHWAY | -0.72 | -2.03 | 0.04 | 0.03 |
| BIOCARTA_CK1_PATHWAY | -0.73 | -2.00 | 0.04 | 0.03 |
| BIOCARTA_GABA_PATHWAY | -0.88 | -2.14 | 0.04 | 0.03 |
| BIOCARTA_NOS1_PATHWAY | -0.82 | -2.40 | 0.04 | 0.03 |
| BIOCARTA_PDZS_PATHWAY | -0.79 | -2.22 | 0.04 | 0.03 |
| BIOCARTA_PGC1A_PATHWAY | -0.83 | -2.22 | 0.04 | 0.03 |
| BIOCARTA_RACC_PATHWAY | -0.82 | -2.18 | 0.04 | 0.03 |
| KEGG_AMYOTROPHIC_LATERAL_SCLEROSIS_ALS | -0.58 | -2.15 | 0.05 | 0.04 |
| KEGG_CARDIAC_MUSCLE_CONTRACTION | -0.54 | -2.10 | 0.06 | 0.05 |
| KEGG_LONG_TERM_DEPRESSION | -0.54 | -2.07 | 0.06 | 0.05 |

| | | | | |
|---|---|---|---|---|
| KEGG_LONG_TERM_POTENTIATION | -0.70 | -2.70 | 0.06 | 0.05 |
| KEGG_PHOSPHATIDYLINOSITOL_SIGNALING_SYSTEM | -0.60 | -2.37 | 0.07 | 0.05 |
| KEGG_SYSTEMIC_LUPUS_ERYTHEMATOSUS | 0.71 | 2.25 | 0.02 | 0.02 |
| PID_AURORA_B_PATHWAY | 0.77 | 2.10 | 0.02 | 0.02 |
| PID_LPA4_PATHWAY | -0.78 | -2.08 | 0.04 | 0.03 |
| PID_PLK1_PATHWAY | 0.73 | 2.03 | 0.02 | 0.02 |
| REACTOME_ACETYLCHOLINE_NEUROTRANSMITTER_RELEASE_CYCLE | -0.89 | -2.52 | 0.04 | 0.03 |
| REACTOME_ACTIVATED_PKN1_STIMULATES_TRANSCRIPTION_OF_AR_ANDROGEN_RECEPTOR_REGULATED_GENES_KLK2_AND_KLK3 | 0.75 | 2.21 | 0.02 | 0.02 |
| REACTOME_ACTIVATION_OF_ANTERIOR_HOX_GENES_IN_HINDBRAIN_DEVELOPMENT_DURING_EARLY_EMBRYOGENESIS | 0.70 | 2.20 | 0.02 | 0.02 |
| REACTOME_ACTIVATION_OF_GENE_EXPRESSION_BY_SREBF_SREBP_ | -0.66 | -2.30 | 0.05 | 0.04 |
| REACTOME_AMINE_LIGAND_BINDING_RECEPTORS | -0.74 | -2.57 | 0.05 | 0.04 |
| REACTOME_AMYLOID_FIBER_FORMATION | 0.65 | 2.01 | 0.02 | 0.02 |
| REACTOME_ANTIGEN_ACTIVATES_B_CELL_RECEPTOR_BCR_LEADING_TO_GENERATION_OF_SECOND_MESSENGERS | 0.68 | 2.06 | 0.02 | 0.02 |
| REACTOME_ASSEMBLY_AND_CELL_SURFACE_PRESENTATION_OF_NMDA_RECEPTORS | -0.61 | -2.15 | 0.05 | 0.04 |
| REACTOME_B_WICH_COMPLEX_POSITIVELY_REGULATES_RRNA_EXPRESSION | 0.69 | 2.08 | 0.0 | 0.0 |

| | | | | |
|---|---|---|---|---|
| | | | 2 | 2 |
| | | | 0.0 | 0.0 |
| REACTOME_BASE_EXCISION_REPAIR | 0.67 | 2.02 | 2 | 2 |
| | | | 0.0 | 0.0 |
| REACTOME_BASE_EXCISION_REPAIR_AP_SITE_FORMATION | 0.71 | 2.08 | 2 | 2 |
| REACTOME_BINDING_AND_UPTAKE_OF_LIGANDS_BY_SCAVENGER_RECEPTO | | | 0.0 | 0.0 |
| RS | 0.74 | 2.27 | 2 | 2 |
| | -0.6 | -2.3 | 0.0 | 0.0 |
| REACTOME_CA_DEPENDENT_EVENTS | 9 | 5 | 5 | 4 |
| | | | 0.0 | 0.0 |
| REACTOME_CD22_MEDIATED_BCR_REGULATION | 0.80 | 2.33 | 2 | 2 |
| | -0.7 | -2.1 | 0.0 | 0.0 |
| REACTOME_CHOLESTEROL_BIOSYNTHESIS | 0 | 6 | 4 | 3 |
| | | | 0.0 | 0.0 |
| REACTOME_CHROMOSOME_MAINTENANCE | 0.64 | 2.03 | 2 | 2 |
| REACTOME_CLASS_C_3_METABOTROPIC_GLUTAMATE_PHEROMONE_RECEPT | -0.5 | -2.0 | 0.0 | 0.0 |
| ORS_ | 9 | 2 | 5 | 4 |
| | | | 0.0 | 0.0 |
| REACTOME_COMPLEMENT_CASCADE | 0.75 | 2.33 | 2 | 2 |
| | | | 0.0 | 0.0 |
| REACTOME_CONDENSATION_OF_PROPHASE_CHROMOSOMES | 0.76 | 2.26 | 2 | 2 |
| | | | 0.0 | 0.0 |
| REACTOME_CREATION_OF_C4_AND_C2_ACTIVATORS | 0.81 | 2.40 | 2 | 2 |
| REACTOME_CREB1_PHOSPHORYLATION_THROUGH_NMDA_RECEPTOR_MEDI | -0.7 | -2.3 | 0.0 | 0.0 |
| ATED_ACTIVATION_OF_RAS_SIGNALING | 4 | 7 | 4 | 3 |
| REACTOME_CYCLIN_A_B1_B2_ASSOCIATED_EVENTS_DURING_G2_M_TRANSIT | | | 0.0 | 0.0 |
| ION | 0.81 | 2.06 | 2 | 2 |
| | -0.7 | -2.5 | 0.0 | 0.0 |
| REACTOME_DAG_AND_IP3_SIGNALING | 2 | 1 | 5 | 4 |

| | | | | |
|---|---|---|---|---|
| REACTOME_DEPOSITION_OF_NEW_CENPA_CONTAINING_NUCLEOSOMES_AT_ | | | 0.0 | 0.0 |
| THE_CENTROMERE | 0.73 | 2.17 | 2 | 2 |
| | | | 0.0 | 0.0 |
| REACTOME_DNA_DAMAGE_TELOMERE_STRESS_INDUCED_SENESCENCE | 0.71 | 2.12 | 2 | 2 |
| | | | 0.0 | 0.0 |
| REACTOME_DNA_METHYLATION | 0.76 | 2.23 | 2 | 2 |
| | -0.9 | -2.7 | 0.0 | 0.0 |
| REACTOME_DOPAMINE_NEUROTRANSMITTER_RELEASE_CYCLE | 0 | 3 | 4 | 3 |
| | -0.7 | -2.3 | 0.0 | 0.0 |
| REACTOME_EFFECTS_OF_PIP2_HYDROLYSIS | 4 | 5 | 4 | 3 |
| REACTOME_ERCC6_CSB_AND_EHMT2_G9A_POSITIVELY_REGULATE_RRNA_E | | | 0.0 | 0.0 |
| XPRESSION | 0.73 | 2.15 | 2 | 2 |
| | | | 0.0 | 0.0 |
| REACTOME_FCERI_MEDIATED_CA_2_MOBILIZATION | 0.70 | 2.10 | 2 | 2 |
| | | | 0.0 | 0.0 |
| REACTOME_FCERI_MEDIATED_MAPK_ACTIVATION | 0.69 | 2.10 | 2 | 2 |
| | | | 0.0 | 0.0 |
| REACTOME_FCGR_ACTIVATION | 0.80 | 2.36 | 2 | 2 |
| | | | 0.0 | 0.0 |
| REACTOME_FCGR3A_MEDIATED_IL10_SYNTHESIS | 0.70 | 2.13 | 2 | 2 |
| REACTOME_FORMATION_OF_THE_BETA_CATENIN_TCF_TRANSACTIVATING_ | | | 0.0 | 0.0 |
| COMPLEX | 0.71 | 2.14 | 2 | 2 |
| | -0.6 | -2.4 | 0.0 | 0.0 |
| REACTOME_G_PROTEIN_MEDIATED_EVENTS | 7 | 8 | 5 | 4 |
| | -0.6 | -2.5 | 0.0 | 0.0 |
| REACTOME_GABA_RECEPTOR_ACTIVATION | 6 | 2 | 6 | 4 |
| | -0.9 | -2.5 | 0.0 | 0.0 |
| REACTOME_GABA_SYNTHESIS_RELEASE_REUPTAKE_AND_DEGRADATION | 0 | 7 | 4 | 3 |
| REACTOME_GLUTAMATE_NEUROTRANSMITTER_RELEASE_CYCLE | -0.9 | -2.7 | 0.0 | 0.0 |

| | | | | |
|---|---|---|---|---|
| | 0 | 3 | 4 | 3 |
| | | | 0.0 | 0.0 |
| REACTOME_HCMV_LATE_EVENTS | 0.67 | 2.08 | 2 | 2 |
| | | | 0.0 | 0.0 |
| REACTOME_HDACS_DEACETYLATE_HISTONES | 0.70 | 2.12 | 2 | 2 |
| REACTOME_IMMUNOREGULATORY_INTERACTIONS_BETWEEN_A_LYMPHOID | | | 0.0 | 0.0 |
| _AND_A_NON_LYMPHOID_CELL | 0.64 | 2.05 | 2 | 2 |
| | | | 0.0 | 0.0 |
| REACTOME_INHIBITION_OF_DNA_RECOMBINATION_AT_TELOMERE | 0.70 | 2.05 | 2 | 2 |
| | | | 0.0 | 0.0 |
| REACTOME_INITIAL_TRIGGERING_OF_COMPLEMENT | 0.80 | 2.38 | 2 | 2 |
| | -0.6 | -2.2 | 0.0 | 0.0 |
| REACTOME_INTERACTION_BETWEEN_L1_AND_ANKYRINS | 8 | 3 | 5 | 3 |
| | -0.6 | -2.4 | 0.0 | 0.0 |
| REACTOME_ION_HOMEOSTASIS | 7 | 6 | 5 | 4 |
| | -0.6 | -2.3 | 0.0 | 0.0 |
| REACTOME_ION_TRANSPORT_BY_P_TYPE_ATPASES | 2 | 0 | 6 | 4 |
| | -0.8 | -2.2 | 0.0 | 0.0 |
| REACTOME_LGI_ADAM_INTERACTIONS | 6 | 3 | 4 | 3 |
| | -0.8 | -2.6 | 0.0 | 0.0 |
| REACTOME_LONG_TERM_POTENTIATION | 6 | 2 | 4 | 3 |
| | | | 0.0 | 0.0 |
| REACTOME_MEIOSIS | 0.65 | 2.03 | 2 | 2 |
| | | | 0.0 | 0.0 |
| REACTOME_MEIOTIC_RECOMBINATION | 0.74 | 2.24 | 2 | 2 |
| | | | 0.0 | 0.0 |
| REACTOME_NEGATIVE_EPIGENETIC_REGULATION_OF_RRNA_EXPRESSION | 0.66 | 2.05 | 2 | 2 |
| REACTOME_NEGATIVE_REGULATION_OF_NMDA_RECEPTOR_MEDIATED_NEU | -0.8 | -2.4 | 0.0 | 0.0 |
| RONAL_TRANSMISSION | 4 | 7 | 4 | 3 |

| | | | | |
|---|---|---|---|---|
| | -0.6 | -2.3 | 0.0 | 0.0 |
| REACTOME_NETRIN_1_SIGNALING | 4 | 7 | 5 | 4 |
| | -0.8 | -2.9 | 0.0 | 0.0 |
| REACTOME_NEUREXINS_AND_NEUROLIGINS | 1 | 9 | 6 | 4 |
| | -0.9 | -2.2 | 0.0 | 0.0 |
| REACTOME_NEUROTOXICITY_OF_CLOSTRIDIUM_TOXINS | 3 | 4 | 4 | 3 |
| | -0.8 | -3.1 | 0.0 | 0.0 |
| REACTOME_NEUROTRANSMITTER_RELEASE_CYCLE | 4 | 1 | 5 | 4 |
| | -0.8 | -2.5 | 0.0 | 0.0 |
| REACTOME_NOREPINEPHRINE_NEUROTRANSMITTER_RELEASE_CYCLE | 9 | 2 | 4 | 3 |
| | | | 0.0 | 0.0 |
| REACTOME_OXIDATIVE_STRESS_INDUCED_SENESCENCE | 0.65 | 2.02 | 2 | 2 |
| | -0.7 | -2.6 | 0.0 | 0.0 |
| REACTOME_PHASE_0_RAPID_DEPOLARISATION | 6 | 3 | 5 | 4 |
| | -0.7 | -2.0 | 0.0 | 0.0 |
| REACTOME_PHASE_4_RESTING_MEMBRANE_POTENTIAL | 3 | 8 | 4 | 3 |
| | -0.6 | -2.0 | 0.0 | 0.0 |
| REACTOME_PLATELET_CALCIUM_HOMEOSTASIS | 4 | 3 | 4 | 3 |
| | | | 0.0 | 0.0 |
| REACTOME_POSITIVE_EPIGENETIC_REGULATION_OF_RRNA_EXPRESSION | 0.65 | 2.00 | 2 | 2 |
| | | | 0.0 | 0.0 |
| REACTOME_PRC2_METHYLATES_HISTONES_AND_DNA | 0.74 | 2.19 | 2 | 2 |
| | | | 0.0 | 0.0 |
| REACTOME_PRE_NOTCH_EXPRESSION_AND_PROCESSING | 0.65 | 2.03 | 2 | 2 |
| REACTOME_PRESYNAPTIC_DEPOLARIZATION_AND_CALCIUM_CHANNEL_OPE | -0.8 | -2.1 | 0.0 | 0.0 |
| NING | 8 | 5 | 4 | 3 |
| REACTOME_RAS_ACTIVATION_UPON_CA2_INFLUX_THROUGH_NMDA_RECEPT | -0.8 | -2.3 | 0.0 | 0.0 |
| OR | 2 | 7 | 4 | 3 |
| REACTOME_RECEPTOR_TYPE_TYROSINE_PROTEIN_PHOSPHATASES | -0.7 | -2.0 | 0.0 | 0.0 |

| | | | | |
|---|---|---|---|---|
| | 3 | 9 | 4 | 3 |
| REACTOME_RECOGNITION_AND_ASSOCIATION_OF_DNA_GLYCOSYLASE_WIT | | | 0.0 | 0.0 |
| H_SITE_CONTAINING_AN_AFFECTED_PURINE | 0.74 | 2.11 | 2 | 2 |
| REACTOME_REGULATION_OF_CHOLESTEROL_BIOSYNTHESIS_BY_SREBP_SRE | -0.6 | -2.2 | 0.0 | 0.0 |
| BF_ | 0 | 2 | 6 | 4 |
| | -0.5 | -2.3 | 0.0 | 0.0 |
| REACTOME_REGULATION_OF_INSULIN_SECRETION | 8 | 1 | 6 | 5 |
| | | | 0.0 | 0.0 |
| REACTOME_RHO_GTPASES_ACTIVATE_PKNS | 0.67 | 2.05 | 2 | 2 |
| | | | 0.0 | 0.0 |
| REACTOME_RMTS_METHYLATE_HISTONE_ARGININES | 0.71 | 2.12 | 2 | 2 |
| | | | 0.0 | 0.0 |
| REACTOME_RNA_POLYMERASE_I_PROMOTER_ESCAPE | 0.70 | 2.13 | 2 | 2 |
| | | | 0.0 | 0.0 |
| REACTOME_RNA_POLYMERASE_I_TRANSCRIPTION | 0.65 | 2.02 | 2 | 2 |
| | | | 0.0 | 0.0 |
| REACTOME_ROLE_OF_LAT2_NTAL_LAB_ON_CALCIUM_MOBILIZATION | 0.75 | 2.22 | 2 | 2 |
| | | | 0.0 | 0.0 |
| REACTOME_ROLE_OF_PHOSPHOLIPIDS_IN_PHAGOCYTOSIS | 0.74 | 2.23 | 2 | 2 |
| REACTOME_RUNX1_REGULATES_GENES_INVOLVED_IN_MEGAKARYOCYTE_D | | | 0.0 | 0.0 |
| IFFERENTIATION_AND_PLATELET_FUNCTION | 0.68 | 2.08 | 2 | 2 |
| REACTOME_RUNX1_REGULATES_TRANSCRIPTION_OF_GENES_INVOLVED_IN_ | | | 0.0 | 0.0 |
| DIFFERENTIATION_OF_HSCS | 0.65 | 2.02 | 2 | 2 |
| | | | 0.0 | 0.0 |
| REACTOME_SCAVENGING_OF_HEME_FROM_PLASMA | 0.80 | 2.35 | 2 | 2 |
| | | | 0.0 | 0.0 |
| REACTOME_SENESCENCE_ASSOCIATED_SECRETORY_PHENOTYPE_SASP_ | 0.71 | 2.19 | 2 | 2 |
| | -0.9 | -2.6 | 0.0 | 0.0 |
| REACTOME_SEROTONIN_NEUROTRANSMITTER_RELEASE_CYCLE | 3 | 1 | 4 | 3 |

| | | | | |
|---|---|---|---|---|
| REACTOME_SEROTONIN_RECEPTORS | -0.86 | -2.16 | 0.04 | 0.03 |
| REACTOME_SIRT1_NEGATIVELY_REGULATES_RRNA_EXPRESSION | 0.75 | 2.21 | 0.02 | 0.02 |
| REACTOME_SYNAPTIC_ADHESION_LIKE_MOLECULES | -0.80 | -2.34 | 0.04 | 0.03 |
| REACTOME_TANDEM_PORE_DOMAIN_POTASSIUM_CHANNELS | -0.81 | -2.02 | 0.04 | 0.03 |
| REACTOME_TRAFFICKING_OF_AMPA_RECEPTORS | -0.79 | -2.60 | 0.05 | 0.03 |
| REACTOME_TRAFFICKING_OF_GLUR2_CONTAINING_AMPA_RECEPTORS | -0.81 | -2.27 | 0.04 | 0.03 |
| REACTOME_TRANSCRIPTIONAL_REGULATION_BY_MECP2 | -0.62 | -2.38 | 0.06 | 0.04 |
| REACTOME_TRANSCRIPTIONAL_REGULATION_BY_SMALL_RNAS | 0.67 | 2.06 | 0.02 | 0.02 |
| REACTOME_TRANSCRIPTIONAL_REGULATION_OF_GRANULOPOIESIS | 0.73 | 2.21 | 0.02 | 0.02 |
| REACTOME_UNBLOCKING_OF_NMDA_RECEPTORS_GLUTAMATE_BINDING_AND_ACTIVATION | -0.86 | -2.52 | 0.04 | 0.03 |
| REACTOME_UPTAKE_AND_ACTIONS_OF_BACTERIAL_TOXINS | -0.65 | -2.10 | 0.04 | 0.03 |
| REACTOME_VOLTAGE_GATED_POTASSIUM_CHANNELS | -0.82 | -2.83 | 0.05 | 0.04 |
| WP_CHOLESTEROL_BIOSYNTHESIS_PATHWAY | -0.77 | -2.07 | 0.04 | 0.03 |
| WP_CHOLESTEROL_METABOLISM_INCLUDES_BOTH_BLOCH_AND_KANDUTSCHRUSSELL_PATHWAYS | -0.60 | -2.15 | 0.05 | 0.04 |
| WP_COMMON_PATHWAYS_UNDERLYING_DRUG_ADDICTION | -0.6 | -2.3 | 0.0 | 0.0 |

| | | | | |
|---|---|---|---|---|
| | 8 | 5 | 5 | 4 |
| | -0.7 | -2.5 | 0.0 | 0.0 |
| WP_DISRUPTION_OF_POSTSYNAPTIC_SIGNALLING_BY_CNV | 6 | 7 | 5 | 3 |
| | -0.8 | -2.7 | 0.0 | 0.0 |
| WP_GABA_RECEPTOR_SIGNALING | 4 | 7 | 5 | 3 |
| | -0.7 | -2.3 | 0.0 | 0.0 |
| WP_MBDNF_AND_PROBDNF_REGULATION_OF_GABA_NEUROTRANSMISSION | 0 | 9 | 5 | 4 |
| | -0.7 | -2.6 | 0.0 | 0.0 |
| WP_MONOAMINE_GPCRS | 8 | 2 | 5 | 3 |
| | -0.5 | -2.0 | 0.0 | 0.0 |
| WP_NOCGMPPKG_MEDIATED_NEUROPROTECTION | 6 | 4 | 5 | 4 |
| | -0.6 | -2.5 | 0.0 | 0.0 |
| WP_PHOSPHODIESTERASES_IN_NEURONAL_FUNCTION | 9 | 4 | 5 | 4 |
| | -0.7 | -2.1 | 0.0 | 0.0 |
| WP_PKCGAMMA_CALCIUM_SIGNALING_PATHWAY_IN_ATAXIA | 2 | 5 | 4 | 3 |
| | -0.5 | -2.0 | 0.0 | 0.0 |
| WP_RENIN_ANGIOTENSIN_ALDOSTERONE_SYSTEM_RAAS | 8 | 5 | 5 | 4 |
| | -0.7 | -2.6 | 0.0 | 0.0 |
| WP_RETT_SYNDROME_CAUSING_GENES | 2 | 3 | 5 | 4 |
| | -0.8 | -2.3 | 0.0 | 0.0 |
| WP_SEROTONIN_AND_ANXIETY | 1 | 0 | 4 | 3 |
| | -0.6 | -2.3 | 0.0 | 0.0 |
| WP_SPLICING_FACTOR_NOVA_REGULATED_SYNAPTIC_PROTEINS | 8 | 8 | 5 | 4 |
| WP_SYNAPTIC_SIGNALING_PATHWAYS_ASSOCIATED_WITH_AUTISM_SPECTR | -0.6 | -2.3 | 0.0 | 0.0 |
| UM_DISORDER | 3 | 4 | 5 | 4 |
| | -0.7 | -2.9 | 0.0 | 0.0 |
| WP_SYNAPTIC_VESICLE_PATHWAY | 8 | 0 | 5 | 4 |
| | | | 0.0 | 0.0 |
| WP_TYROBP_CAUSAL_NETWORK | 0.69 | 2.01 | 2 | 2 |

| | | | | |
|---|---|---|---|---|
| | -0.6 | -2.1 | 0.0 | 0.0 |
| WP_VITAMIN_DSENSITIVE_CALCIUM_SIGNALING_IN_DEPRESSION | 3 | 7 | 5 | 4 |
