# Supplementary material for: Prognostic value and immune-infiltration pattern of FOXD3-AS1 in patients with glioma
Source: Front Pharmacol. 2023 Apr 4;14:1162309. doi: 10.3389/fphar.2023.1162309 (PMC10110859; doi:10.3389/fphar.2023.1162309)
Supplement: Supplementary file 8 [file Table3.pdf]

Supplementary Table 3. Univariate and multivariate Cox regression analysis of FOXD3-AS1 combined with clinical variables

Characteristics	Total	Univariate analysis		Multivariate analysis	
		HR(95% CI)	P	HR(95% CI)	P
WHO grade	634				
G2	223	Reference			
G3	243	2.999 (2.007-4.480)	<0.001	1.695 (1.055-2.723)	0.029
G4	168	18.615 (12.460-27.812)	<0.001	4.607 (1.412-15.027)	0.011
1p/19q codeletion	688				
codel	170	Reference			
non-codel	518	4.428 (2.885-6.799)	<0.001	1.347 (0.755-2.404)	0.314
Primary therapy outcome	461				
PD	112	Reference			
SD	147	0.440 (0.294-0.658)	<0.001	0.416 (0.250-0.693)	<0.001
PR	64	0.170 (0.074-0.391)	<0.001	0.211 (0.075-0.594)	0.003
CR	138	0.133 (0.064-0.278)	<0.001	0.174 (0.081-0.375)	<0.001
IDH status	685				
WT	246	Reference			
Mut	439	0.117 (0.090-0.152)	<0.001	0.587 (0.349-0.988)	0.045
Gender	695				
Female	297	Reference			
Male	398	1.262 (0.988-1.610)	0.062	1.830 (1.171-2.860)	0.008
Age	695				
<=60	552	Reference			
>60	143	4.668 (3.598-6.056)	<0.001	4.544 (2.733-7.553)	<0.001
Histological type	695				

Astrocytoma&Glioblastoma	363	Reference			
Oligoastrocytoma&Oligodendroglioma	332	0.267 (0.204-0.350)	<0.001	0.902 (0.574-1.416)	0.653
FOXD3-AS1	695				
Low	347	Reference			
High	348	3.884 (2.972-5.075)	<0.001	2.079 (1.332-3.243)	0.001
