# Supplementary material for: Prognostic value and immune-infiltration pattern of FOXD3-AS1 in patients with glioma
Source: Front Pharmacol. 2023 Apr 4;14:1162309. doi: 10.3389/fphar.2023.1162309 (PMC10110859; doi:10.3389/fphar.2023.1162309)
Supplement: Supplementary file 9 [file Table7.pdf]

Supplementary Table 7. Correlation between RHCG and immune cells in the LUAD microenvironment

ID	Immune cells	Cor(Pearson)	P(Pearson)
FOXD3-AS1	Th2 cells	0.433	<0.001
	Tcm	-0.355	<0.001
	Macrophages	0.335	<0.001
	Eosinophils	0.308	<0.001
	TFH	-0.285	<0.001
	Tem	-0.280	<0.001
	NK CD56bright cells	-0.268	<0.001
	Neutrophils	0.260	<0.001
	aDC	0.211	<0.001
	TReg	-0.209	<0.001
